# Supplementary material for: Mathematical modelling to estimate the impact of maternal and perinatal healthcare services and interventions on health in sub-Saharan Africa: A scoping review
Source: PLoS One. 2024 Dec 2;19(12):e0296540. doi: 10.1371/journal.pone.0296540 (PMC11611201; doi:10.1371/journal.pone.0296540)
Supplement: S2 File — (DOCX) [file pone.0296540.s002.docx]

In part A each study title is provided alongside a study number. This number is used to identify reviewed studies in parts B-D.

*Data extraction form (part A) – Study characteristics*

| **Study N^o^** | **Study title** | **First author**  **(year)** | **Sub-Saharan African countries modelled** | **Single country study?** | **Affiliation country of first author** | **First author affiliation in study country?** | **Any author affiliation in study country?** | **Study evaluates cost?** | **Notes** |
| --- | --- | --- | --- | --- | --- | --- | --- | --- | --- |
| **1** | *Cost-effectiveness of antenatal multiple micronutrients and balanced energy protein supplementation compared to iron and folic acid supplementation in India, Pakistan, Mali, and Tanzania: A dynamic microsimulation study* | Young et al. (2022) | *Mali and Tanzania* | *No* | *USA* | *No* | *No* | *Yes* | *Additionally, Pakistan and India were modelled.* |
| **2** | *Mobile Technology for Community Health in Ghana: Is Maternal Messaging and Provider Use of Technology Cost-Effective in Improving Maternal and Child Health Outcomes at Scale?* | Willcox et al. (2019) | *Ghana* | *Yes* | *USA* | *No* | *Yes* | *Yes* | *Modelling was conducted to as part of wider cost-effectiveness study* |
| **3** | *Effective coverage and budget implications of skill-mix change to improve neonatal nursing care: an explorative simulation study in Kenya* | Tsiachristas et al. (2019) | *Kenya* | *Yes* | *UK* | *No* | *Yes* | *No* | *Study also includes elicitation workshop with stakeholders to partially inform the model.* |
| **4** | *Cost-Effectiveness of Interventions to Improve Maternal, Newborn and Child Health Outcomes: A WHO-CHOICE Analysis for Eastern Sub-Saharan Africa and South-East Asia* | Stenberg et al. (2021) | *Burundi, Comoros, Djibouti, Eritrea, Ethiopia, Kenya, Madagascar, Malawi, Mauritius, Mozambique, Rwanda, Somalia, South Sudan, Sudan, Uganda, Tanzania, Zambia* | *No* | *Switzerland* | *No* | *No* | *Yes* | *Additionally, 11 countries in southeast Asia were modelled.* |
| **5** | *Applying a system dynamics modelling approach to explore policy options for improving neonatal health in Uganda* | Semwanga et al. (2016) | *Uganda* | *Yes* | *Uganda* | *Yes* | *Yes* | *No* | *N/A* |
| **6** | *Predicted effect of regionalised delivery care on neonatal mortality, utilisation, financial risk, and patient utility in Malawi: an agent-based modelling analysis* | Shrime et al. (2019) | *Malawi* | *Yes* | *USA* | *No* | *Yes* | *No* | *N/A* |
| **7** | *Estimation of potential effects of improved community-based drug provision, to augment health-facility strengthening, on maternal mortality due to post-partum haemorrhage and sepsis in sub-Saharan Africa: an equity-effectiveness model* | Pagel et al. (2009) | *Malawi* | *Yes* | *UK* | *No* | *Yes* | *No* | *N/A* |
| **8** | *Predicting resource-dependent maternal health outcomes at a referral hospital in Zanzibar using patient trajectories and mathematical modelling* | Nadkarni et al. (2019) | *Zanzibar* | *Yes* | *USA* | *No* | *No* | *No* | *N/A* |
| **9** | *Triple return on investment: the cost and impact of 13 interventions that could prevent stillbirths and save the lives of mothers and babies in South Africa* | Michalow et al. (2015) | *South Africa* | *Yes* | *South Africa* | *Yes* | *Yes* | *Yes* | *N/A* |
| **10** | *A cost-effectiveness analysis of maternal and neonatal health interventions in Ethiopia* | Memirie et al. (2019) | *Ethiopia* | *Yes* | *Ethiopia* | *Yes* | *Yes* | *Yes* | *N/A* |
| **11** | *Cost-effectiveness of community-based practitioner programmes in Ethiopia, Indonesia and Kenya* | McPake et al. (2015) | *Ethiopia, Indonesia, Kenya* | *No* | *Australia* | *No* | *Yes* | *Yes* | *N/A* |
| **12** | *Tranexamic Acid to Reduce Postpartum Hemorrhage: A MANDATE Systematic Review and Analyses of Impact on Maternal Mortality* | McClure et al. (2015) | *Sub-Saharan Africa* | *No* | *USA* | *No* | *No* | *No* | *The region of sub-Saharan Africa is modelled – regional inputs are used (i.e., average facility delivery rate, average PPH incidence)* |
| **13** | *Projecting the lives saved by continuing the historical scale-up of child and maternal health interventions in Mozambique until 2030* | Júnior et al. (2019) | *Mozambique* | *Yes* | *Mozambique* | *Yes* | *No* | *No* | *N/A* |
| **14** | *Forecasting the Value for Money of Mobile Maternal Health Information Messages on Improving Utilization of Maternal and Child Health Services in Gauteng, South Africa: Cost-Effectiveness Analysis* | LeFevre et al. (2018) | *South Africa* | *Yes* | *South Africa* | *Yes* | *No* | *No* | *N/A* |
| **15** | *Cost-effectiveness of community health systems strengthening: quality improvement interventions at community level to realise maternal and child health gains in Kenya* | Kumar et al. (2021) | *Kenya* | *Yes* | *Kenya* | *Yes* | *Yes* | *Yes* | *N/A* |
| **16** | *Newborn Survival Case Study in Rwanda - Bottleneck Analysis and Projections in Key Maternal and Child Mortality Rates Using Lives Saved Tool (LiST)* | Khurmi et al. (2017) | *Rwanda* | *Yes* | *Rwanda* | *Yes* | *No* | *No* | *N/A* |
| **17** | *Resuscitation and Obstetrical Care to Reduce Intrapartum-Related Neonatal Deaths: A MANDATE Study* | Kamath-Rayne et al. (2015) | *sub-Saharan Africa* | *No* | *USA* | *No* | *No* | *No* | *The region of sub-Saharan Africa is modelled – regional inputs are used. In addition, India is modelled.* |
| **18** | *Using the lives saved tool (LiST) to model mHealth impact on neonatal survival in resource-limited settings* | Jo et al. (2014) | *Uganda* | *No* | *USA* | *No* | *No* | *No* | *Additionally, Bangladesh is modelled.* |
| **19** | *A low-cost uterine balloon tamponade for management of postpartum hemorrhage: modeling the potential impact on maternal mortality and morbidity in sub-Saharan Africa* | Herrick et al. (2017) | *sub-Saharan Africa* | *No* | *USA* | *No* | *No* | *No* | *The region of sub-Saharan Africa is modelled – regional inputs are used* |
| **20** | *Interventions to reduce neonatal mortality: a mathematical model to evaluate impact of interventions in sub-Saharan Africa* | Griffin et al. (2017) | *sub-Saharan Africa* | *No* | *USA* | *No* | *No* | *No* | *The region of sub-Saharan Africa is modelled – regional inputs are used* |
| **21** | *Evaluating WHO-Recommended Interventions for Preterm Birth: A Mathematical Model of the Potential Reduction of Preterm Mortality in Sub-Saharan Africa* | Griffin et al. (2019) | *sub-Saharan Africa* | *No* | *USA* | *No* | *No* | *No* | *The region of sub-Saharan Africa is modelled – regional inputs are used* |
| **22** | *Reducing maternal mortality from preeclampsia and eclampsia in low-resource countries - what should work?* | Goldenberg et al. (2015) | *sub-Saharan Africa* | *No* | *USA* | *No* | *No* | *No* | *The region of sub-Saharan Africa is modelled – regional inputs are used* |
| **23** | *Clinical interventions to reduce stillbirths in sub-Saharan Africa: a mathematical model to estimate the potential reduction of stillbirths associated with specific obstetric conditions.* | Goldenberg et al. (2018) | *sub-Saharan Africa* | *No* | *USA* | *No* | *No* | *No* | *The region of sub-Saharan Africa is modelled – regional inputs are used* |
| **24** | *Sub-Saharan Africa's Mothers, Newborns, and Children: How Many Lives Could Be Saved with Targeted Health Interventions?* | Friberg et al. (2010) | *Cameroon, Ethiopia, Ghana, Kenya, Nigeria, Senegal, South Africa, Tanzania, Uganda and additional 33 countries in sub-Saharan Africa (see notes)* | *No* | *USA* | *Yes* | *Yes* | *No* | *33 sub-Saharan African countries not listed in text or supplementary material. The countries Cape Verde, Mauritius, Sao Tome and Principe, and Seychelles were not modelled due to low births.* |
| **25** | *Estimating the cost and cost-effectiveness for obstetric fistula repair in hospitals in Uganda: a low income country* | Epiu et al. (2018) | *Uganda* | *Yes* | *Uganda* | *Yes* | *Yes* | *Yes* | *N/A* |
| **26** | *Evaluation of the Ethiopian Millennium Rural Initiative: impact on mortality and cost-effectiveness* | Curry et al. (2013) | *Ethiopia* | *Yes* | *USA* | *No* | *Yes* | *Yes* | *N/A* |
| **27** | *Cost and impact of scaling up interventions to save lives of mothers and children: taking South Africa closer to MDGs 4 and 5* | Chola et al. (2015) | *South Africa* | *Yes* | *South Africa* | *Yes* | *Yes* | *Yes* | *N/A* |
| **28** | *Cost-Effectiveness of Peer Counselling for the Promotion of Exclusive Breastfeeding in Uganda* | Chola et al. (2015) | *Uganda* | *Yes* | *South Africa* | *No* | *Yes* | *Yes* | *N/A* |
| **29** | *Cost-effectiveness of inhaled oxytocin for prevention of postpartum haemorrhage: a modelling study applied to two high burden settings* | Carvalho et al. (2020) | *Ethiopia* | *No* | *Australia* | *No* | *No* | *Yes* | *Additionally, Bangladesh was modelled.* |
| **30** | *Evidence-based interventions for improvement of maternal and child nutrition: What can be done and at what cost?* | Bhutta et al. (2013) | *Nigeria, Ethiopia, The Democratic Republic of the Congo (DRC), Sudan, Kenya, Uganda, Mozambique, Madagascar, Niger, Malawi, South Africa, Cote D’Ivoire and Burkina Faso* | *No* | *Pakistan* | *Yes* | *Yes* | *No* | *Additionally, India, Pakistan, Indonesia, Bangladesh, The Philippines, Egypt, Vietnam, Yemen, Nepal, Myanmar, Iraq, and Guatemala were modelled.* |
| **31** | *The impact and cost of scaling up midwifery and obstetrics in 58 low- and middle-income countries* | Bartlett et al. (2014) | *Benin, Botswana, Burkina Faso, Burundi, Cameroon, Central African Republic, Chad, Comoros, Cote d’ivoire, Democratic Republic of the Congo, Djibouti, Ethiopia, Gabon, Gambia, Ghana, Guinea, Guinea-Bissau, Kenya, Liberia, Madagascar, Malawi, Mali, Mauritania, Mozambique, Myanmar, Nigeria, Niger, Rwanda, Senegal, Sierra Leone, Somalia, South Africa, Sudan, Tanzania, Togo, Uganda, Zambia, Zimbabwe* | *No* | *USA* | *No* | *No* | *Yes* | *Additionally, Afghanistan, Bangladesh, Bhutan, Bolivia, Cambodia, East Timor, Guyana, Haiti, Indonesia, Madagascar, Nepal, Nicaragua, Tajikistan, Vietnam, India, Lao PDR, Morocco, Myanmar, Pakistan, Papua New Guinea, Uzbekistan, and Yemen were modelled.* |
| **32** | *Cost effectiveness of a novel device for improving resuscitation of apneic newborns* | Ali et al. (2020) | *Tanzania* | *Yes* | *USA* | *No* | *No* | *Yes* | *N/A* |
| **33** | *Promotion of WHO feeding recommendations: a model evaluating the effects on HIV-free survival in African children* | David et al. (2008) | *Benin, Botswana, Burkina Faso, Burundi, Cameroon, Central African Republic, Chad, Eritrea, Ethiopia, Gabon, Ghana, Guinea, Ivory Coast,  Kenya, Liberia, Malawi, Mali, Mauritania, Mozambique, Namibia, Niger, Nigeria, Rwanda, Senegal, South Africa, Tanzania, Togo, Uganda, Zambia, Zimbabwe* | *No* | *France* | *No* | *No* | *No* | *Additionally, Sudan was modelled.* |
| **34** | *Saving newborn lives in Asia and Africa: cost and impact of phased scale-up of interventions within the continuum of care* | Darmstadt et al. (2008) | *Angola, Benin, Botswana, Burkina Faso, Burundi, Cameroon, Central African Republic, Chad, Congo, Cote d’Ivoire, Democratic Republic of Congo, Djibouti, Equatorial Guinea, Ethiopia, Gabon, Gambia, Ghana, Guinea, Guinea-Bissau, Kenya, Liberia, Malawi, Mali, Mauritania, Madagascar, Mozambique, Niger,  Nigeria, Rwanda, Senegal, Sierra Leone, Somalia, South Africa, Tanzania, Togo, Uganda, Zambia, and Zimbabwe* | *No* | *USA* | *No* | *No* | *Yes* | *Additionally, Pakistan, Bangladesh, India, Nepal, Azerbaijan, Tajikistan, Turkmenistan, Kazakhstan, Cambodia, China, Indonesia, Myanmar, Papua New Guinea, the Philippines, Brazil, Haiti, Mexico, Iraq, Yemen, Sudan, Swaziland, and Afghanistan were modelled.* |
| **35** | *Cost-effectiveness of misoprostol to control postpartum hemorrhage in low-resource settings.* | Bradley et al. (2007) | *Tanzania* | *Yes* | *USA* | *No* | *No* | *Yes* | *N/A* |
| **36** | *The potential of medical abortion to reduce maternal mortality in Africa: what benefits for Tanzania and Ethiopia?* | Baggaley et al. (2010) | *Tanzania and Ethiopia* | *No* | *UK* | *No* | *No* | *No* | *N/A* |
| **37** | *Estimating the global impact of poor quality of care on maternal and neonatal outcomes in 81 low- and middle-income countries: A modeling study* | Chou et al. (2019) | *Angola, Benin, Botswana, Burkina Faso, Burundi, Cameroon, Central African Republic, Chad, Comoros, Congo, DRC, Côte d'Ivoire, Djibouti, Equatorial Guinea, Eritrea, Ethiopia, Gabon, Gambia, Ghana, Guinea, Guinea-Bissau, Kenya, , Lesotho, Liberia, Madagascar, Malawi, Mali Mauritania, Mozambique,, Namibia, Niger, Nigeria, Rwanda, Senegal, Sierra Leone, Somalia, South Africa, South Sudan, Tanzania, Uganda, Zambia, Zimbabwe* | *No* | *USA* | *No* | *No* | *No* | *Additionally, Afghanistan, Algeria, Azerbaijan, Bangladesh, Bhutan, Bolivia, Cambodia, Dominican Republic, Equatorial Guinea, Guatemala, Guinea, Guyana, Haiti, Honduras, India, Indonesia, Iraq, Jamaica, Korea, Kyrgyz Republic, Lao, Morocco, Myanmar, Nepal, Nicaragua, Pakistan, Panama, Papua New Guinea, Paraguay, Philippines, Solomon Islands, Sudan, Suriname, Swaziland, Timor-Leste, Togo, Turkmenistan, Uzbekistan, Venezuela, and Yemen were modelled* |
| **38** | *Potential impact of midwives in preventing and reducing maternal and neonatal mortality and stillbirths: a Lives Saved Tool modelling study* | Nove et al. (2021) | *Angola, Benin, Botswana, Burkina Faso, Burundi, Cameroon, Central African Republic, Chad, , Comoros, Congo, Côte d'Ivoire, The Democratic Republic of the Congo, Djibouti, Equatorial Guinea, Eritrea, Eswatini, Ethiopia, Gabon, Gambia, Ghana, Guinea, Guinea-Bissau, Kenya, Lesotho, Liberia, Madagascar, Malawi, Mali, Mauritania, Mozambique, Namibia, Niger, Nigeria, Rwanda, São Tomé and Príncipe, Senegal, Sierra Leone, Somalia, South Africa, South Sudan, Uganda, Tanzania, Zambia, Zimbabwe* | *No* | *UK* | *No* | *Yes* | *No* | *Additionally, Afghanistan, Algeria, Azerbaijan, Bangladesh, Bhutan, Bolivia, Brazil, Cambodia, China, DPR Korea, Dominican Republic, Egypt, Guatemala, Guyana, Haiti, Honduras, India, Indonesia, Iraq, Jamaica, Kyrgyzstan, Lao PDR, Mexico, Morocco, Myanmar, Nepal, Nicaragua, Pakistan, Panama, Papua New Guinea, Paraguay, Peru, Philippines, Solomon Islands, Sudan, Suriname, Tajikistan, Timor-Leste, Togo, Turkmenistan,, Uzbekistan, Venezuela, Vietnam, Yemen were modelled.* |
| **39** | *Maternal Mortality from Obstructed Labor: A MANDATE Analysis of the Ability of Technology to Save Lives in Sub-Saharan Africa* | Harrison et al. (2016) | *sub-Saharan Africa* | *No* | *USA* | *No* | *No* | *No* | *The region of sub-Saharan Africa is modelled – regional inputs are used* |
| **40** | *Incremental cost and health gains of the 2016 WHO antenatal care recommendations for Rwanda: results from expert elicitation* | Hitimana et al. (2019) | *Rwanda* | *Yes* | *Rwanda* | *Yes* | *Yes* | *Yes* | *N/A* |
| **41** | *Improving maternal and newborn care: cost-effectiveness of an innovation to rebrand traditional birth attendants in Sierra Leone* | Fotso et al. (2020) | *Sierra Leone* | *Yes* | *Cameroon* | *No* | *No* | *Yes* | *N/A* |
| **42** | *Assessing health and economic outcomes of interventions to reduce pregnancy-related mortality in Nigeria* | Erim et al. (2012) | *Nigeria* | *Yes* | *USA* | *No* | *No* | *Yes* | *N/A* |
| **43** | *Strategic planning for saving the lives of mothers, newborns and children and preventing stillbirths in KwaZulu-Natal province South Africa: modelling using the Lives Saved Tool (LiST)* | McGee et al. (2016) | *South Africa* | *Yes* | *South Africa* | *Yes* | *Yes* | *Yes* | *N/A* |
| **44** | *Using the Lives Saved Tool to aid country planning in meeting mortality targets: a case study from Mali* | Keita et al. (2017) | *Mali* | *Yes* | *USA* | *No* | *Yes* | *No* | *N/A* |
| **45** | *Expanding the population coverage of evidence-based interventions with community health workers to save the lives of mothers and children: an analysis of potential global impact using the Lives Saved Tool (LiST)* | Chou et al. (2017) | *Angola, Benin, Botswana, Burkina Faso, Burundi, Cameroon, Central African Republic, Chad, Comoros, Congo, Côte d'Ivoire, Democratic Republic of the Congo, Djibouti, Equatorial Guinea, Eritrea, Ethiopia, Gabon, Gambia, Ghana, Guinea, Guinea-Bissau, Kenya, Lesotho, Liberia, Madagascar, Malawi, Mali, Mauritania, Mozambique, Niger, Nigeria, Rwanda, São Tomé and Príncipe, Senegal, Sierra Leone, Somalia, South Africa, South Sudan, Swaziland, Togo, Uganda, United Republic of Tanzania, Zambia, Zimbabwe* | *No* | *USA* | *No* | *No* | *No* | *Additionally, Bolivia, Brazil, Guatemala, Haiti, Peru, Bangladesh, Dem. People's Republic of Korea, India, Indonesia, Myanmar, Nepal, Azerbaijan, Kyrgyzstan, Tajikistan, Turkmenistan, Uzbekistan, Afghanistan, Egypt, Iraq, Morocco, Pakistan, Sudan, Yemen, Cambodia, Lao, Papua New Guinea, Philippines, Solomon Islands, Viet Nam were modelled.* |
| **46** | *The projected effect of scaling up midwifery* | Homer et al. (2014) | *Benin, Burkina Faso, Burundi, Central African Republic, Chad, Comoros, Cote d’Ivoire, Democratic Republic of the Congo, Djibouti, Eritrea, Ethiopia, Gambia, Guinea, Guinea-Bissau, Liberia, Malawi, Mali, Mozambique, Niger, Rwanda, Sierra Leone, Somalia, Sudan, Zambia, Zimbabwe, Angola, Cameroon, Congo, Kenya, Lesotho, Madagascar, Mauritania, Nigeria, Sao Tome and Principe, Senegal, Tanzania, Togo, Uganda, Yemen, Botswana, Equatorial Guinea, Gabon, Ghana, South Africa* | *No* | *Australia* | *No* | *No* | *No* | *Afghanistan, Sudan, Bangladesh, Bhutan, Cambodia, Cameroon, Congo, Haiti, Laos, Myanmar, Nepal, Pakistan, Papua New Guinea, Solomon Islands, Swaziland, Timor-Leste, Yemen, Azerbaijan, Bolivia, Brazil, China, Egypt, Guatemala, Guyana, India, Indonesia, Iraq, Kyrgyzstan, North Korea, Mexico, Morocco, Nicaragua, Peru, Philippines, Tajikistan, Turkmenistan, Uzbekistan, and Vietnam were modelled.* |
| **47** | *Stillbirths: what difference can we make and at what cost?* |  | *Angola, Benin, Botswana, Burkina Faso, Burundi, Cameroon, Central African Republic, Chad, , Congo, Côte d’Ivoire, Djibouti, DR Congo, Equatorial Guinea, Eritrea, Ethiopia, Gabon, Ghana, Guinea, Guinea-Bissau, Kenya, Lesotho, Liberia, Madagascar, Malawi, Mali, Mauritania, Mozambique, Niger, Nigeria, Rwanda, Senegal, Sierra Leone, Somalia, South Africa, Tanzania, The Gambia, Togo, Uganda, Zambia, Zimbabwe* | *No* | *Pakistan* | *Yes* | *Yes* | *Yes* | *Additionally, Afghanistan, Azerbaijan, Bangladesh, Bolivia, Brazil, Cambodia, Cameroon, China, Egypt, Guatemala, Haiti, India, Indonesia, Iraq, Laos, Mexico, Morocco, Myanmar, Nepal, North Korea, Pakistan, Papua New Guinea, Peru, Philippines, Sudan, Swaziland, Tajikistan, Turkmenistan and Yemen were modelled.* |
| **48** | *Using LiST to model potential reduction in under-five mortality in Burkina Faso* | Bhutta et al. (2011) | *Burkina Faso* | *Yes* | *USA* | *No* | *Yes* | *No* | *N/A* |
| **49** | *Addressing inequity to achieve the maternal and child health millennium development goals: looking beyond averages* | Marsh et al. (2013) | *Tanzania* | *Yes* | *Tanzania* | *Yes* | *Yes* | *No* | *N/A* |
| **50** | *Prioritizing child health interventions in Ethiopia: modeling impact on child mortality, life expectancy and inequality in age at death* | Ruhago et al. (2012) | *Ethiopia* | *Yes* | *Norway* | *Yes* | *Yes* | *No* | *N/A* |
| **51** | *Pushing the envelope through the Global Financing Facility: potential impact of mobilising additional support to scale-up life-saving interventions for women, children and adolescents in 50 high-burden countries* | Onarheim et al. (2012) | *Cameroon, Central Africa Republic, Cote, D’Ivoire, Democratic Republic Congo, Ethiopia, Guinea, Kenya, Liberia, Madagascar, Malawi, Mali, Mozambique, Nigeria, Rwanda, Senegal, Sierra Leone, Tanzania, Uganda* | *No* | *USA* | *No* | *No* | *Yes* | *I was unable to find a full list of the fifty modelled countries in the manuscript or the supplementary material. Therefore, I have only extracted those countries mentioned in-text.*  *In addition to the sub-Saharan African countries Afghanistan, Bangladesh, Cambodia, Guatemala, Haiti, Indonesia, Myanmar, and Vietnam were modelled.* |
| **52** | *Patterns in coverage of maternal, newborn, and child health interventions: projections of neonatal and under-5 mortality to 2035* | Chou et al. (2018) | *Angola, Benin, Botswana, Burkina Faso, Burundi, Cameroon, Cape Verde, Central African Republic, Chad, Comoros, Congo, Congo, Dem. Rep., Cote d'Ivoire, Djibouti, Equatorial Guinea, Eritrea, Ethiopia, Gabon, Gambia, Ghana, Guinea, Guinea-Bissau, Kenya, Lesotho, Liberia, Madagascar, Malawi, Mali, Mauritania, Mozambique, Namibia, Niger, Nigeria, Rwanda, Sao Tome and Principe, Senegal, Sierra Leone, Somalia, South Africa, Swaziland, Tanzania, Togo, Uganda, Zambia, Zimbabwe* | *No* | *USA* | *No* | *No* | *No* | *Additionally, Afghanistan, Albania, Algeria, Armenia, Azerbaijan, Bangladesh, Belarus, Belize, Bolivia, Bosnia and Herzegovina, , Brazil,  Cambodia, China, Colombia, Cuba, Dominican Republic, Egypt, Georgia, Guatemala, Guinea, Guyana, Haiti, Honduras, India, Indonesia, Iraq, Jamaica, Jordan, Kazakhstan, Korea, Dem. Rep., Kyrgyzstan, Lao, Lebanon, Macedonia, Maldives, Mexico, Moldova, Mongolia, Montenegro, Morocco, Myanmar, Nepal, Nicaragua, Pakistan, Papua, New Guinea, Peru, Philippines, Sudan, Suriname, Thailand, Timor-Leste, Trinidad, and Tobago, Tunisia, Turkey, Turkmenistan, Ukraine, Uzbekistan, Vanuatu, Venezuela, Vietnam and Yemen were modelled.* |
| **53** | *How Ethiopia achieved Millennium Development Goal 4 through multisectoral interventions: a Countdown to 2015 case study* | Walker et al. (2013) | *Ethiopia* | *Yes* | *USA* | *No* | *Yes* | *No* | *N/A* |
| **54** | *Malawi and Millennium Development Goal 4: a Countdown to 2015 country case study* | Ruducha et al. (2017) | *Malawi* | *Yes* | *Malawi* | *Yes* | *Yes* | *No* | *N/A* |
| **55** | *Progress and priorities for reproductive, maternal, newborn, and child health in Kenya: A Countdown to 2015 country case study* | Kanyuka et al. (2016) | *Kenya* | *Yes* | *Canada* | *No* | *Yes* | *No* | *N/A* |
| **56** | *Tanzania's countdown to 2015: an analysis of two decades of progress and gaps for reproductive, maternal, newborn, and child health, to inform priorities for post-2015* | Keats et al. (2017) | *Tanzania* | *Yes* | *UK* | *No* | *Yes* | *No* | *N/A* |
| **57** | *Intervention heroes of Mozambique from 1997 to 2015: estimates of maternal and child lives saved using the Lives Saved Tool* | Afnan-Holmes et al. (2015) | *Mozambique* | *Yes* | *Mozambique* | *Yes* | *Yes* | *No* | *N/A* |
| **58** | *Explaining progress towards Millennium Development Goal 4 for child survival in Tanzania* | Macicame et al. (2018) | *Tanzania* | *Yes* | *Tanzania* | *Yes* | *Yes* | *No* | *N/A* |
| **59** | *Reduction in child mortality in Niger: a Countdown to 2015 country case study* | Niyeha et al. (2018) | *Niger* | *Yes* | *USA* | *No* | *Yes* | *No* | *N/A* |
| **60** | *A simulation-based comparative effectiveness analysis of policies to improve global maternal health outcomes* | Ward et al. (2023) | *Angola, Benin, Botswana, Burkina Faso, Burundi, Cabo Verde, Cameroon, Central African Republic, Chad, Comoros, Congo, Côte d’Ivoire, Democratic Republic of the Congo, Djibouti, Equatorial Guinea, Eritrea, Ethiopia, Gabon, Gambia, Ghana, Guinea, Guinea-Bissau, Kenya, Lesotho, Liberia, Madagascar, Malawi, Mali, Mauritania Mauritius, Mozambique, Namibia, Niger, Nigeria, Rwanda, Sao Tome and Principe, Senegal, Seychelles, Sierra Leone, Somalia, South Africa , South Sudan, Uganda, United Republic of Tanzania, Zambia, Zimbabwe* | *No* | *USA* | *No* | *Yes* | *No* | *Additionally, Afghanistan, Albania, Algeria, Andorra, Antigua and Barbuda, Argentina, Armenia, Australia, Austria, Azerbaijan, Bahama, Bahrain, Bangladesh, Barbados, Belarus, Belgium, Belize, Bermuda, Bhutan, Bolivia, Bosnia, and, Herzegovina, Brazil, Brunei, Bulgaria, Cambodia, Canada, Cayman, Islands, Chile, China, Colombia, Costa, Rica, Croatia, Cuba, Cyprus, Czechia, Dem People’s Republic of Korea, Denmark, Dominica, Dominican, Republic, Ecuador, Egypt, El, Salvador, Estonia, Faroe Islands, Fiji, Finland, France, Georgia, Germany, Greece, Greenland, Grenada, Guatemala, Guyana, Haiti, Honduras, Hungary, Iceland, India, Indonesia, Iran (Islamic Republic of), Iraq, Ireland, Israel, Italy, Jamaica, Japan, Jordan, Kazakhstan, Kiribati, Kuwait, Kyrgyzstan, Lao People’s Democratic Republic, Latvia, Lebanon, Libya, Liechtenstein, Lithuania, Luxembourg, Madagascar, Malaysia, Maldives, Malta, Marshall Islands, Mexico, Micronesia (Fed States of), Monaco, Mongolia, Montenegro, Morocco, Myanmar, Nauru, Nepal, Netherlands, New Zealand, Nicaragua, North Macedonia, Norway, Oman, Pakistan, Palau, Panama, Papua, New Guinea, Paraguay, Peru, Philippines, Poland, Portugal, Puerto, Rico, Qatar, Republic of Korea, Republic of Moldova, Romania, Russian, Federation, Saint Kitts and Nevis, Saint Lucia, Saint Vincent and the Grenadines, Samoa, San Marino, Saudi Arabia, Serbia, Seychelles, Singapore, Slovakia, Slovenia, Solomon Islands, Spain, Sri Lanka, State of Palestine, Sudan, Suriname, Sweden, Switzerland, Syrian, Arab, Republic, Taiwan, Tajikistan, Thailand, Timor-Leste, Togo, Tonga, Trinidad, and, Tobago, Tunisia, Türkiye, Turkmenistan, Tuvalu, Ukraine, United Arab Emirates, United Kingdom, United Republic of Tanzania, United States of America, Uruguay, Uzbekistan, Vanuatu, Venezuela (Bolivarian Republic of), Viet Nam, and Yemen were modelled* |

*Data extraction form (part B) – Intervention characteristics*

| **Study N^o^** | **Interventions (IVs) evaluated**^[[1]](#footnote-1)^ | **Additional notes on IVs?** | **Any novel IVs?** | **IVs delivered to women?** | **If yes – when?** | **IVs delivered to neonates?** | **Are effects on maternal outcomes of interest?** | **Are effects on neonatal outcomes of interest?** | **Are effects on stillbirths of interest?** |
| --- | --- | --- | --- | --- | --- | --- | --- | --- | --- |
| **1** | 1. Multiple micronutrient supplementation during pregnancy 2. Balanced energy protein supplementation during pregnancy | *N/A* | *No* | *Yes* | *Antenatal* | *No* | *No* | *Yes* | *No* |
| **2** | 1. The Mobile Technology for Community Health (MOTECH) Initiative 2. Skilled birth attendants 3. Facility delivery 4. Measles immunisation | *Effect of MOTECH program estimated using lives saved tool. MOTECH was found to increase coverage of other listed interventions via propensity score analysis.* | *Yes* | *Yes* | *Antenatal Intrapartum Postpartum* | *Yes* | *Yes* | *Yes* | *Yes* |
| **3** | 1. Impact of introducing neonatal healthcare assistants in neonatal nursing care | *A number of alternative “skill-mix” scenarios were modelled in which staff-to-newborn ratio was varied.* | *No* | *No* | *N/A* | *Yes* | *No* | *Yes* | *No* |
| **4** | 1. Family planning 2. Folic acid supplementation 3. Safe abortion services 4. Post abortion case management 5. Calcium supplementation in pregnant women for the prevention and management of pre-eclampsia/eclampsia 6. Daily iron and folic acid supplementation in pregnant women 7. Balanced energy-protein supplementation to pregnant women living in areas with high food insecurity balance 8. Tetanus toxoid vaccination 9. Intermittent presumptive treatment of malaria in pregnancy 10. Syphilis detection and treatment in pregnancy 11. Hypertensive disease case management in pregnancy 12. Management of pre-eclampsia (mild and severe) 13. Ectopic pregnancy case management 14. Neonatal resuscitation 15. Clean cord care (clean birth practices) 16. Antibiotics for preterm premature rupture of membranes 17. Management of eclampsia with magnesium-sulphate 18. Management of maternal sepsis 19. Promotion of breastfeeding 20. Home visits for clean postnatal practice 21. Vitamin A supplementation (0-4 years) 22. Promotion of complementary feeding 23. Diphtheria, Pertussis, Tetanus (DPT) vaccine 24. Haemophilus influenzae type b (Hib) vaccine 25. Pneumococcal vaccine 26. Rotavirus vaccine 27. Pentavalent vaccine (DPT + Hepatitis B + Hib) 28. Measles vaccine 29. Kangaroo mother care 30. Full supportive care for premature babies 31. Case management of severe neonatal infection (sepsis/pneumonia) with full supportive care 32. Facility-based management of neonatal infection (sepsis/pneumonia) with injectable (and oral) antibiotics 33. Management of diarrhoea through oral rehydration solution and zinc 34. Community-based management of pneumonia 35. Antibiotics for treatment of dysentery 36. Facility-based management of pneumonia 37. Management of children with severe acute malnutrition | *N/A* | *No* | *Yes* | *Antenatal Intrapartum Postpartum* | *Yes* | *Yes* | *Yes* | *No* |
| **5** | 1. Community health education 2. Motorcycle ambulance coupons 3. Free delivery kits 4. Community health education + free delivery kits 5. Community health education + motorcycle ambulance coupons 6. Motorcycle ambulance coupons + free delivery kit 7. Kangaroo mother care 8. Skills in neonatal resuscitation 9. Skills in labour management 10. Malaria prevention 11. Anaemia prevention 12. Tetanus toxoid prevention | *N/A* | *No* | *Yes* | *Antenatal*  *Intrapartum* | *No* | *Yes* | *Yes* | *No* |
| **6** | 1. Restricted deliveries to facilities currently capable of providing CSs 2. Restricted deliveries to facilities currently capable of providing CSs but with selected facilities upgraded to provide CSs 3. Restricted delivery to facilities that provided five or more basic emergency obstetric and neonatal care services in the preceding 3 months 4. Restricted delivery to facilities that provided five or more basic emergency obstetric and neonatal care services in the preceding 3 months but with selected facilities upgraded to provide at least five basic emergency obstetric and neonatal care services | *Modelled ‘interventions’ were varied scenarios of regionalised delivery. Arguably could be defined as one intervention.* | *No* | *Yes* | *Intrapartum* | *Yes* | *Yes* | *Yes* | *No* |
| **7** | 1. Health facilities are provided with uterotonics and antibiotics 2. Health facilities are provided with uterotonics and antibiotics with (i) distribution of misoprostol to women attending outreach ANC and (ii) distribution of antibiotics by community health workers to women with signs of postnatal sepsis 3. Health facilities are provided with uterotonics and antibiotics with (i) distribution of misoprostol to women attending outreach ANC and (ii) distribution of antibiotics by community health workers to women with signs of postnatal sepsis with additional access to misoprostol and antibiotics via female volunteers in villages | *Authors also define these interventions as packages – “1) health-facility strengthening; 2) health-facility strengthening combined with improved drug provision via antenatal-care appointments and community health workers; and 3) all interventions in package two combined with improved community-based drug provision via female volunteers in villages”* | *No* | *Yes* | *Postpartum* | *No* | *Yes* | *No* | *No* |
| **8** | 1. Improved/varied availability of essential medicines within the ward environment - oxytocin, hydralazine (increased stocking) 2. Varied number of doctors and nurses available to deliver care | *N/A* | *No* | *Yes* | *Antenatal Intrapartum Postpartum* | *No* | *Yes* | *No* | *No* |
| **9** | 1. Syphilis detection and treatment 2. Hypertensive disease case management 3. Diabetes case management 4. Magnesium sulphate (MgSO_4_) Management of pre-eclampsia 5. Foetal growth restriction detection and management 6. Labour and delivery management 7. Induction of labour for pregnancies lasting 41+ weeks 8. Early detection and treatment of HIV in pregnant women 9. Tetanus toxoid immunization during pregnancy 10. Antibiotics for preterm premature rupture of membranes 11. Antenatal corticosteroids for preterm labour 12. Active management of the third stage of labour 13. Neonatal resuscitation | *N/A* | *No* | *Yes* | *Antenatal Intrapartum Postpartum* | *Yes* | *Yes* | *Yes* | *Yes* |
| **10** | 1. Safe abortion 2. Tetanus toxoid 3. Syphilis detection and treatment 4. Calcium supplementation 5. Management of preeclampsia and eclampsia 6. Antibiotics for preterm Premature Rupture of Membranes (pPROM) 7. Antenatal corticosteroids for preterm labour 8. Active management of the third stage of labour 9. Induction of labour (beyond 41 weeks) 10. Maternal sepsis case management 11. Neonatal resuscitation (institutional) 12. Neonatal sepsis 13. Kangaroo mother care | *N/A* | *No* | *Yes* | *Antenatal Intrapartum Postpartum* | *Yes* | *Yes* | *Yes* | *No* |
| **11** | 1. Community based practitioners programme 2. Antenatal care 3. Tetanus toxoid administration 4. Iron folate supplementation 5. Skilled birth attendance 6. Promotion of breastfeeding 7. Preventive postnatal care 8. Hygienic disposal of children’s faeces 9. Household ownership of Insecticide-treated bed nets (ITNs) 10. BCG vaccine 11. Polio vaccine 12. DPT vaccine 13. Measles vaccine | *Effect of community-based practitioners programme estimated using the Lives Saved Tool by increasing coverage of the other listed interventions.* | *No* | *Yes* | *Antenatal Intrapartum Postpartum* | *Yes* | *Yes* | *Yes* | *Yes* |
| **12** | 1. Tranexamic acid | *N/A* | *No* | *Yes* | *Postpartum* | *No* | *Yes* | *No* | *No* |
| **13** | 1. Any breastfeeding (6-11 months) 2. Any breastfeeding (12-23 months) 3. Artemisinin-based combination therapies (ACTs) for malaria 4. BCG vaccine 5. Care-seeking for pneumonia 6. DPT3 vaccine 7. Exclusive breastfeeding (0-5 months) 8. Facility delivery 9. Hepatitis B vaccine 10. Improved drinking water source 11. Improved sanitary infrastructure 12. ITNs 13. Intermittent preventive treatment of malaria in pregnancy (IPTp) 14. Measles vaccine 15. Oral rehydration solution (ORS) for diarrhoea 16. Prenatal care: 4+ visits 17. Polio vaccine 18. Safe disposal of child’s faeces 19. Skilled birth attendant 20. Tetanus vaccine in pregnancy: 2+ doses 21. Vitamin A (for children 6-59 months) 22. Water piped inside the household | *N/A* | *No* | *Yes* | *Antenatal Intrapartum Postpartum* | *Yes* | *Yes* | *Yes* | *No* |
| **14** | 1. Mobile Alliance for Maternal Action (MAMA) program 2. ANC4+ 3. Full immunisation | *MAMA program is a text based Mobile Maternal Health Information Message system. Effect of the programme estimated using the Lives Saved Tool by increasing coverage of other listed interventions. Immunisations are not listed in the text.* | *Yes* | *Yes* | *Antenatal Postpartum* | *No* | *Yes* | *Yes* | *No* |
| **15** | 1. Community-level Quality Improvement health system strengthening intervention | *Intervention improves quality of ANC and facility delivery services which are assumed to reduce mortality.* | *Yes* | *Yes* | *Antenatal Intrapartum* | *No* | *Yes* | *Yes* | *No* |
| **16** | 1. Tetanus toxoid vaccination 2. IPTp or ITN 3. Syphilis detection and treatment 4. Calcium supplementation 5. Micronutrient supplementation 6. Balanced energy supplementation 7. Hypertensive disorder case management 8. Diabetes case management 9. Malaria case management 10. Magnesium sulphate management of pre-eclampsia 11. Foetal growth restriction detection and management 12. Skilled birth attendance 13. Exclusive breastfeeding at 1 month 14. Clean postnatal practices 15. Chlorhexidine 16. Hand washing with soap 17. Maternal Sepsis case management 18. Case management of premature babies 19. Oral antibiotics for neonatal sepsis 20. Oral antibiotics for neonatal sepsis | *N/A* | *No* | *Yes* | *Antenatal Intrapartum Postpartum* | *Yes* | *Yes* | *Yes* | *No* |
| **17** | 1. Diagnosis: recognition of need for resuscitation 2. Treatment: manual stimulation 3. Treatment: resuscitation/bag mask ventilation 4. Diagnosis: home recognition of intrapartum-related injury 5. Diagnosis: clinical diagnosis of intrapartum-related injury 6. Treatment: oxygen, pulse oximetry, antibiotic 7. Treatment: continuous positive airway pressure 8. Treatment: neonatal intensive care unit/ventilation 9. Transfer with recognition/diagnosis of need for post-resuscitation care 10. BEmONC 11. CEmONC | *Broadly, the authors group interventions as basic neonatal resuscitation and advance neonatal care. BEmONC AND CEmONC interventions modelled for delivery to mothers are not listed separately.* | *No* | *Yes* | *Intrapartum Postpartum* | *Yes* | *No* | *Yes* | *No* |
| **18** | 1. Antenatal care 2. Skilled birth attendance and/or Facility delivery 3. Breastfeeding promotion 4. Postnatal care 5. BEmONC 6. CEmONC | *‘Targets’ for mHealth strategies identified using LiST- supported by evidence that mHealth improves service use* | *No* | *Yes* | *Antenatal Intrapartum Postpartum* | *Yes* | *No* | *Yes* | *No* |
| **19** | 1. Uterine Balloon Tamponade | *N/A* | *No* | *Yes* | *Postpartum* | *No* | *Yes* | *No* | *No* |
| **20** | 1. Recognition of asphyxiated infants 2. All deliveries in hospital with clean delivery practices, chlorhexidine cord care and antibiotics 3. Antibiotics with high recognition of neonatal infection at home, clinic, and hospitals 4. Bed nets and IPTp and deliveries in a hospital with high recognition of neonatal malaria with transfer; improved neonatal antimalarial treatment 5. Bed nets and IPTp for all pregnancies with current level of hospital care 6. Chlorhexidine cord care for all newborns at home, clinic, and hospitals 7. Clean delivery practices at home, clinic, and hospital 8. Clean delivery practices, chlorhexidine cord care and antibiotics at home, clinics, and hospitals 9. Diagnosis and maternal transfer for ante- and intrapartum haemorrhage, preeclampsia/eclampsia obstructed labour, and foetal distress plus recognition and resuscitation of asphyxiated infants, with transfer and treatment with oxygen, Continuous Positive Airway Pressure (CPAP), and ventilation, if necessary and treatment (including caesarean) for key obstetric complications 10. High level ANC and hospital delivery for all pregnancies with current level of obstetric and neonatal hospital care 11. High neonatal tetanus recognition: transfer, improved neonatal treatment 12. High recognition of neonatal malaria with neonatal transfer to hospitals 13. High recognition of neonatal malaria with transfer, and improved antimalarial treatment 14. High recognition of neonatal syphilis at home, clinics and hospitals with transfer and current care 15. High recognition of neonatal syphilis at home, clinics and hospitals with transfer and improved antibiotic treatment 16. High recognition of neonatal tetanus, with transfer, current levels of neonatal care 17. Hospital care for all pregnancies with appropriate treatment for ante- and intrapartum haemorrhage, obstructed labour, preeclampsia/eclampsia, and foetal distress including CS and resuscitation and appropriate neonatal care for asphyxiated infants 18. Hospital delivery for all pregnancies 19. Hospital delivery for all pregnancies with current obstetric/neonatal care 20. Hospital delivery for all pregnancies with recognition and resuscitation of asphyxiated infants, with transfer and treatment with oxygen, CPAP, and ventilation, if necessary 21. Maternal syphilis screening and treatment in all locations and all deliveries in hospitals with high recognition of neonatal syphilis and improved antibiotic treatment 22. Maternal syphilis screening, treatment, and high recognition of syphilis at home, clinics and hospitals with transfer and improved antibiotic treatment 23. Preterm deliveries in a hospital with maternal treatment with corticosteroids, high recognition of respiratory distress and treatment, including surfactants, oxygen, CPAP, and newborn intensive care 24. Recognition and resuscitation of asphyxiated infants, with transfer and treatment with oxygen, CPAP, and ventilation 25. Recognition and transfer of mother for ante- and intrapartum haemorrhage, preeclampsia/eclampsia obstructed labour and foetal distress with current levels of hospital care plus recognition and resuscitation of asphyxiated infants, with transfer and treatment with oxygen, CPAP, and ventilation, if necessary 26. Recognition of asphyxiated infants and resuscitation of asphyxiated infants 27. Recognition of impending preterm birth and maternal transfer to hospitals with maternal treatment with corticosteroids and preterm infant treatment with surfactants, oxygen, CPAP, and newborn intensive care 28. Recognition of impending preterm birth and transfer to hospitals 29. Recognition of impending preterm birth, transfer, and antenatal corticosteroids in hospitals 30. Recognition of neonatal respiratory distress and treatment, including surfactants, oxygen, CPAP and newborn intensive care in clinics and hospitals 31. Recognition of preterm birth, transfer, of sick neonate with current levels of care 32. Recognition of respiratory distress, transfer and oxygen in clinics and hospitals for infants with RDS 33. Tetanus prevention with vaccination, clean cord care, and clean delivery 34. Tetanus prevention with vaccination, clean cord care, and clean delivery practices, high recognition of neonatal tetanus with transfer, and improved neonatal treatments | *N/A* | *No* | *Yes* | *Antenatal Intrapartum Postpartum* | *Yes* | *No* | *Yes* | *No* |
| **21** | 1. Antenatal corticosteroids 2. Antibiotics for preterm labour 3. Cord care 4. Thermal care for preterm newborns 5. Feeding 6. Immediate drying and additional stimulation 7. Positive Pressure Ventilation (PPV) 8. Oxygen therapy for preterm newborns 9. Continuous positive airway pressure for newborns with respiratory distress syndrome 10. Surfactant administration for newborns with RDS 11. Prophylactic antibiotics for prevention of sepsis 12. Empirical antibiotics for suspected neonatal sepsis 13. Antibiotics for treatment of necrotising enterocolitis |  | *No* | *Yes* | *Antenatal Intrapartum* | *Yes* | *No* | *Yes* | *No* |
| **22** | 1. Blood pressure measurement 2. Detection of proteinuria 3. Antihypertensives 4. Magnesium sulfate 5. CS/induction for prevention/treatment of eclampsia/death) 6. Transfer (for mild/moderate preeclampsia) 7. Transfer (for severe preeclampsia and eclampsia) |  | *No* | *Yes* | *Antenatal Intrapartum Postpartum* | *No* | *Yes* | *No* | *No* |
| **23** | 1. All women deliver in hospital with improved diagnosis and CS early enough to maximise foetal outcomes 2. All women deliver in hospital; improved BP/proteinuria; foetal distress monitoring; early CS 3. All women deliver in hospital, improved foetal monitoring. 4. All women deliver in hospital; improved partograph diagnosis of obstructed labour (OL); CS early 5. All women deliver in hospital; improved ultrasound diagnosis of foetal-growth restriction (FGR) (third trimester); foetal distress monitoring; CS early enough to maximise foetal outcomes 6. All women deliver in hospitals with current level of treatment 7. CS early enough in clinical course to maximise foetal outcomes 8. CS early enough to maximise foetal outcomes 9. CS or operative delivery early to maximise foetal outcomes 10. Deliver in hospital with current care 11. Early CS or operative delivery in hospital 12. Improved antibiotic treatment for maternal syphilis 13. Improved bed nets 14. Improved blood pressure (BP)/proteinuria diagnosis of pre-eclampsia (PE) 15. Improved BP/proteinuria diagnosis of PE; transfer to clinic or hospital 16. Improved BP/proteinuria diagnosis of PE; transfer to hospital, foetal distress monitoring, early CS 17. Improved CS sufficiently early to impact the foetus 18. Improved diagnosis of antepartum haemorrhage (APH); transfer to clinic or hospital 19. Improved diagnosis of malaria, with transfer to clinic or hospital 20. Improved early diagnosis of APH 21. Improved early diagnosis of APH with transfer to clinic or hospital 22. Improved foetal monitoring in clinics and hospital 23. Improved foetal monitoring in clinics w/transfer to hospital 24. Improved foetal monitoring in clinics/hospitals; transfer to hospital; CS or early operative delivery 25. Improved IPT Improved IPT and bed nets 26. Improved IPT, bed nets and maternal malaria diagnosis; transfer, antimalarial treatment for maternal malaria 27. Improved malaria diagnosis, transfer, and antimalarial treatment 28. Improved partograph diagnosis of OL 29. Improved partograph diagnosis of OL with transfer to clinic or hospital 30. Improved screening and antibiotic treatment for maternal syphilis 31. Improved screening for maternal syphilis 32. Improved ultrasound diagnosis for conditions and foetal monitoring 33. Improved ultrasound diagnosis of FGR (third trimester) in all settings 34. Improved ultrasound diagnosis of FGR (third trimester) in all settings; transfer 35. Improved ultrasound diagnosis of FGR (third trimester) in all settings; transfer to clinic or hospital; foetal distress monitoring in hospital; CS early to maximise foetal outcomes 36. Improved ultrasound diagnosis of FGR (third trimester) in all settings; transfer to hospital; foetal distress monitoring in hospital 37. Ultrasound diagnosis of conditions, foetal distress monitoring and transfer 38. Vaginal operative delivery in hospitals |  | *No* | *Yes* | *Antenatal Intrapartum* | *No* | *No* | *No* | *Yes* |
| **24** | 1. Comprehensive Emergency Obstetric Care 2. Calcium supplementation 3. Antibiotics for pPROM 4. Clean practices and immediate essential newborn care (home) 5. Essential care for all women and immediate essential newborn care (facility) 6. Basic Emergency Obstetric Care 7. Active management of the third state of labour 8. Ectopic pregnancy case management (BEmOC level) 9. Ectopic pregnancy case management (CEmOC level) 10. IPTp 11. Tetanus toxoid immunization 12. Oral rehydration salts (ORS) 13. Syphilis detection and treatment 14. Preventive postnatal care (healthy practices & illness detection) 15. Oral antibiotic case management of severe infection 16. Injectable antibiotic case management of severe infection 17. Case management of severe infection with full supportive care 18. Essential care for all women and immediate essential newborn care 19. Neonatal resuscitation (institutional) 20. Neonatal resuscitation (home) 21. Antenatal corticosteroids for preterm labour 22. Kangaroo mother care 23. Case management of severe illness with full supportive care 24. Periconceptual Folic Acid 25. Case management of serious neonatal illness 26. Use of improved water source within 30 minutes 27. Use of water connection in the home 28. Improved excreta disposal (latrine/toilet) 29. Hand washing with soap 30. Hygienic disposal of children's stool 31. Vitamin A for prevention 32. Zinc for prevention 33. Rotavirus vaccine 34. Antibiotics for dysentery 35. Zinc for treatment 36. Hib vaccine 37. Pneumococcal vaccine 38. DPT vaccination 39. Case management of pneumonia (oral antibiotics) 40. Measles vaccine 41. Vitamin A for measles treatment 42. Insecticide treated materials/indoor residual spraying 43. Antimalarials |  | *No* | *Yes* | *Antenatal Intrapartum Postpartum* | *Yes* | *Yes* | *Yes* | *No* |
| **25** | 1. Surgical repair of obstetric fistula |  | *No* | *Yes* | *Postpartum* | *No* | *Yes* | *No* | *No* |
| **26** | 1. Antenatal care (ANC) 2. Skilled birth attendance 3. Postnatal care 4. HIV testing during ANC 5. Measles vaccination coverage 6. Pentavalent 3 vaccine coverage |  | *No* | *Yes* | *Antenatal Intrapartum Postpartum* | *Yes* | *Yes* | *Yes* | *No* |
| **27** | 1. Safe abortion services 2. Post abortion case management 3. Ectopic pregnancy case management 4. Antenatal care (4 visits) 5. Tetanus toxoid vaccination 6. Calcium supplementation 7. Hypertensive disease case management 8. Diabetes case management 9. MgSO4 10. management of pre-eclampsia 11. Foetal growth restriction detection and management 12. Skilled birth attendance (SBA) 13. Facility delivery (clinic and hospital) 14. Unassisted deliveries 15. BEMOC 16. CEMOC 17. Clean birth practices 18. Immediate assessment and stimulation 19. Labour and delivery management 20. Neonatal resuscitation 21. Antenatal corticosteroids for preterm labour 22. Antibiotics for pPROM 23. MgSO_4_ management of eclampsia 24. Active management of the third stage of labour 25. Induction of labour for pregnancies lasting 41-weeks 26. Promotion of breastfeeding 27. Preventive postnatal care 28. Clean postnatal practices 29. Complementary feeding - education only 30. Complementary feeding-supplementation and education 31. Vitamin A supplementation 32. Improved water source 33. Water connection in the home 34. Improved sanitation-utilisation of latrines or toilets 35. Hand washing with soap 36. Hygienic disposal of children’s stools 37. Polio vaccine 38. DPT vaccine 39. Hib vaccine 40. Hep vaccine 41. Pneumococcal vaccine 42. Rotavirus vaccine 43. Measles vaccine 44. Maternal Sepsis case management 45. Kangaroo mother care 46. Case management of severe neonatal infection 47. Injectable antibiotics 48. Full supportive care 49. ORS 50. Antibiotics-for treatment of dysentery 51. Zinc-for treatment of diarrhoea 52. Oral antibiotics: case management of pneumonia in children 53. Vitamin A-for treatment of measles 54. Therapeutic feeding-for severe wasting 55. Treatment for moderate acute malnutrition 56. Treatment of childhood injuries 57. Treatment of childhood TB 58. Early detection and treatment of HIV in pregnant women 59. Inter-facility transport |  | *No* | *Yes* | *Antenatal Intrapartum Postpartum* | *Yes* | *Yes* | *Yes* | *No* |
| **28** | 1. Community-based peer counselling conducted alongside breastfeeding promotion in facility-based maternal and child health services, including antenatal and postnatal service |  | *Yes* | *Yes* | *Antenatal Postpartum* | *No* | *No* | *Yes* | *No* |
| **29** | 1. Inhaled oxytocin product |  | *Yes* | *Yes* | *Postpartum* | *No* | *Yes* | *No* | *No* |
| **30** | 1. Periconceptional folic acid supplementation or fortification 2. Maternal balanced energy protein supplementation 3. Maternal calcium supplementation 4. Multiple micronutrient supplementation in pregnancy 5. Promotion of breast feeding 6. Appropriate complementary feeding 7. Vitamin A and preventive zinc supplementation in children 6–59 months of age, 8. Management of severe acute malnutrition (SAM) 9. Management of moderate acute malnutrition (MAM) |  | *No* | *Yes* | *Antenatal Postpartum* | *Yes* | *Yes* | *Yes* | *No* |
| **31** | 1. Family planning services 2. Safe abortion services 3. Post abortion care 4. Tetanus toxoid vaccination 5. Syphilis detection and treatment 6. Multiple micronutrient supplementation 7. Calcium supplementation 8. Diabetes screening and management 9. Prevention & management of malaria 10. Hypertension screening & management 11. Detection of foetal growth 12. Clean birth practices 13. Immediate assessment of neonate 14. MGSO^4^ 15. Active management of the third stage of labour (AMTSL) 16. Antibiotics for pPROM 17. Induction of post-term labour 18. Residual skilled delivery (BEmONC) 19. Residual skilled delivery with C-section 20. Clean postnatal practices 21. Thermal care 22. Kangaroo mother care 23. Exclusive breastfeeding promotion 24. Maternal sepsis case management |  | *No* | *Yes* | *Antenatal Intrapartum Postpartum* | *Yes* | *Yes* | *Yes* | *Yes* |
| **32** | 1. The Augmented Infant Resuscitator |  | *Yes* | *No* | *N/A* | *Yes* | *No* | *Yes* | *No* |
| **33** | 1. Exclusive breastfeeding for 6 months 2. Replacement feeding with 3 promotion strategies |  | *No* | *Yes* | *Postpartum* | *Yes* | *No* | *Yes* | *No* |
| **34** | 1. Folic acid supplementation 2. Tetanus toxoid immunisation 3. Syphilis screening and treatment 4. Prevention of pre-eclampsia and eclampsia 5. IPT for malaria 6. Detection and treatment of bacteriuria 7. Antibiotics for pPROM 8. Corticosteroids for preterm labour 9. Detection and management of breech, multiple pregnancy 10. Labour surveillance (including partograph) for early diagnosis of complications 11. Clean birth practices 12. Newborn resuscitation 13. Breastfeeding 14. Prevention and management of hypothermia 15. Kangaroo mother care (Low birth weight infants in health facilities) 16. Community based pneumonia case management |  | *No* | *Yes* | *Antenatal Intrapartum Postpartum* | *Yes* | *No* | *Yes* | *No* |
| **35** | 1. Misoprostol delivered by traditional birth attendant to treat PPH |  | *No* | *Yes* | *Postpartum* | *No* | *Yes* | *No* | *No* |
| **36** | 1. Medical abortion with misoprostol |  | *No* | *Yes* | *Antenatal* | *No* | *Yes* | *No* | *No* |
| **37** | 1. Tetanus toxoid vaccination 2. Intermittent preventive treatment of malaria during pregnancy 3. Syphilis detection and treatment 4. Iron supplementation in pregnancy 5. Hypertensive disorder case management 6. Diabetes case management 7. Malaria case management 8. MgSO4 management of preeclampsia 9. Clean birth practices 10. Labour and delivery management 11. Neonatal resuscitation 12. Antibiotics for pPROM 13. Management of eclampsia with MgSO4 14. AMTSL 15. Induction of labour for pregnancies lasting 41+ weeks 16. Hygienic cord care 17. Thermal care for case management of premature babies 18. Kangaroo Mother Care for case management of premature babies 19. Case management of neonatal sepsis/pneumonia with injectable antibiotics |  | *No* | *Yes* | *Antenatal Intrapartum Postpartum* | *Yes* | *Yes* | *Yes* | *Yes* |
| **38** | 1. Modern methods of contraception 2. Folic acid supplementation 3. Ectopic pregnancy care management 4. Safe abortion services 5. Post abortion care 6. Tetanus toxoid vaccine 7. Intermittent preventive treatment in pregnancy 8. Syphilis detection and treatment, if needed 9. Iron folate supplementation 10. Hypertension screening and management 11. Diabetes case management 12. Malaria case management 13. Screening for and management of pre-eclampsia with magnesium sulphate 14. Prevention of mother-to-child transmission of HIV 15. Clean birth environment 16. Immediate drying and additional stimulation 17. Thermal protection 18. Clean cord care 19. Manual removal of placenta 20. Parenteral administration of anti-convulsant 21. Antibiotics for preterm or prolonged PROM 22. Parenteral administration of antibiotics 23. Assisted vaginal delivery 24. Neonatal resuscitation 25. Parenteral administration of uterotonics 26. Removal of retained products of conception 27. Induction of labour for pregnancies lasting 41+weeks 28. Antenatal corticosteroids for preterm labour 29. Breastfeeding promotion 30. Kangaroo mother care 31. Injectable antibiotics for neonatal sepsis |  | *No* | *Yes* | *Antenatal Intrapartum Postpartum* | *Yes* | *Yes* | *Yes* | *Yes* |
| **39** | 1. Clinical decision making 2. Decision making with partogram 3. Prolonged/obstructed labour (POL) in the first/second stage of labour treated by CS 4. POL in the second stage of labour treated by operative vaginal delivery (OVD) 5. Transfer of setting |  | *No* | *Yes* | *Intrapartum* | *No* | *Yes* | *No* | *No* |
| **40** | 1. ANC8 for normal (uncomplicated) pregnancies |  | *No* | *Yes* | *Antenatal* | *No* | *Yes* | *Yes* | *Yes* |
| **41** | 1. Essential Newborn Care Corps intervention 2. ANC4+ 3. Health facility delivery 4. Postpartum care, mothers 5. Postnatal care, newborns 6. Breastfeeding <1 hour 7. Iron supplementation |  | *Yes* | *Yes* | *Antenatal Intrapartum Postpartum* | *Yes* | *Yes* | *Yes* | *No* |
| **42** | 1. Contraception 2. Safe abortion and post-abortion care 3. Prevention and treatment of anaemia (including intermittent prevention and treatment of malaria in pregnancy) 4. Intrapartum care 5. Postpartum care |  | *No* | *Yes* | *Antenatal Intrapartum Postpartum* | *Yes* | *Yes* | *No* | *No* |
| **43** | 1. Folic acid supplementation/fortification 2. Safe abortion services 3. Post abortion case management 4. Ectopic pregnancy case management 5. Antenatal care 6. Tetanus toxoid vaccination 7. Syphilis detection and treatment 8. Calcium supplementation 9. Iron folate supplementation 10. Multiple micronutrient supplementation 11. Balanced energy supplementation 12. Hypertensive disease case management 13. Diabetes case management 14. MgSO_4_ - Management of pre-eclampsia 15. FGR detection and management 16. Skilled birth attendance 17. Facility delivery (clinic and hospital) 18. Home deliveries 19. Unassisted deliveries 20. Assisted deliveries at home 21. Facility deliveries 22. Essential care 23. Basic Emergency Obstetric Care 24. Comprehensive Emergency Obstetric Care 25. Clean birth practices 26. Immediate assessment and stimulation 27. Labour and delivery management 28. Neonatal resuscitation 29. Antenatal corticosteroids for preterm labour 30. Antibiotics for pPROM 31. MgSO4 management of eclampsia 32. AMTSL 33. Induction of labour for pregnancies lasting 41+ weeks 34. Promotion of breastfeeding 35. Preventive postnatal care 36. Thermal care 37. Clean postnatal practices 38. Chlorhexidine 39. Complementary feeding–education only 40. Complementary feeding–supplementation and education 41. Vitamin A supplementation 42. Zinc supplementation 43. Improved water source 44. Water connection in the home 45. Improved sanitation - Utilization of latrines or toilets 46. Hand washing with soap 47. Hygienic disposal of children’s stools 48. BCG vaccine 49. Polio vaccine 50. DPT vaccine 51. Hib vaccine 52. HepB vaccine 53. Pneumococcal vaccine 54. Rotavirus vaccine 55. Measles vaccine 56. Maternal Sepsis case management 57. Kangaroo mother care 58. Case management of severe neonatal infection 59. Oral antibiotics 60. Injectable antibiotics 61. Full supportive care 62. ORS 63. Antibiotics - for treatment of dysentery 64. Zinc - for treatment of diarrhoea 65. Oral antibiotics: case management of pneumonia in children 66. Vitamin A - for treatment of measles 67. Antimalarials - Artemisinin compounds for malaria 68. Therapeutic feeding - for severe wasting 69. Treatment for moderate acute malnutrition |  | *No* | *Yes* | *Antenatal Intrapartum Postpartum* | *Yes* | *Yes* | *Yes* | *Yes* |
| **44** | 1. Contraceptive prevalence 2. Antenatal care 3. Tetanus toxoid vaccination 4. Pregnant women protected by insecticide treated bed nets (ITN) 5. Iron Supplementation 6. Malaria case management 7. Skilled birth attendance 8. Health facility delivery 9. BEmOC 10. Exclusive breastfeeding (0 – 1 month) 11. Exclusive breastfeeding (1 – 5 month) 12. Clean postnatal practices 13. Vitamin A supplementation 14. Zinc supplementation 15. Improved water source 16. Water connection in the home 17. Utilization of latrines or toilets 18. Hand washing with soap 19. Ownership of ITNs 20. DPT-three doses 21. HiB vaccine – three doses 22. HepB – three doses 23. Measles vaccine – single dose 24. BCG vaccine – single dose 25. Rotavirus vaccine -two doses 26. Pneumococcal vaccine – three doses 27. Polio vaccine – three doses 28. Thermal care 29. Oral antibiotic for newborn 30. Vitamin A for Measles treatment 31. Newborn sepsis case management 32. ORS – oral rehydration solution 33. Antibiotic for treatment of dysentery 34. Zinc – for treatment of diarrhoea 35. Oral antibiotic for pneumonia 36. Artemisinin for malaria 37. Stunting 38. Wasting |  | *No* | *Yes* | *Antenatal Intrapartum Postpartum* | *Yes* | *Yes* | *Yes* | *Yes* |
| **45** | 1. Folic acid via supplementation or fortification 2. Tetanus toxoid immunization 3. IPTp 4. Calcium supplementation 5. Syphilis detection and treatment 6. Micronutrient supplementation 7. Balanced energy and protein supplementation 8. Skilled birth attendance 9. Clean birth practices 10. Immediate assessment and stimulation 11. Neonatal resuscitation 12. Promotion of breastfeeding 13. Clean postnatal practices 14. Chlorhexidine umbilical cord treatment 15. Thermal care 16. Oral antibiotics for neonatal sepsis 17. Education and provision of complementary foods 18. Vitamin A supplementation 19. Zinc supplementation 20. Immunization for vaccine-preventable diseases 21. Hand washing with soap 22. Safe disposal of children's stools 23. Use of insecticide-treated nets or indoor residual spraying 24. Oral rehydration solution for childhood diarrhoea 25. Zinc for treatment of diarrhoea 26. Antibiotics for dysentery 27. Oral antibiotics for pneumonia 28. Treatment for moderate acute malnutrition 29. Vitamin A for treatment of measles 30. Artemisinin compounds for malaria treatment |  | *No* | *Yes* | *Antenatal Intrapartum* | *Yes* | *Yes* | *Yes* | *Yes* |
| **46** | 1. Contraceptive Prevalence Rate 2. Folic acid supplementation 3. Ectopic pregnancy case management 4. Safe abortion services 5. Post abortion care 6. Tetanus toxoid 7. IPTp 8. Multiple micronutrient supplementation 9. Calcium supplementation 10. Balanced energy supplementation 11. Syphilis detection and treatment if needed 12. Diabetes case management 13. Screening for and management of preeclampsia with MgSO_4_ 14. Case management of malaria in pregnancy 15. Screening and management of foetal growth restriction 16. Prevention of mother to child transmission (PMTCT) 17. Clean birth practices 18. Immediate assessment and stimulation 19. Skilled attendant at birth 20. Neonatal resuscitation 21. Antenatal corticosteroids 22. Antibiotics for pPROM 23. MgSO_4_ for eclampsia 24. AMTSL 25. Induction of post-term labour 26. Thermal care and clean postnatal practices 27. Kangaroo mother care 28. Maternal sepsis case management 29. Breastfeeding promotion 30. Hospital based care for severe newborn infections |  | *No* | *Yes* | *Antenatal Intrapartum Postpartum* | *Yes* | *Yes* | *Yes* | *Yes* |
| **47** | 1. Periconceptional folic acid fortification 2. Prevention of malaria with insecticide-treated bed nets or intermittent preventive treatment with antimalarials 3. Syphilis detection and treatment 4. Detection and management of hypertensive disease of pregnancy 5. Detection and management of diabetes of pregnancy 6. Detection and management of foetal growth restriction (including caesarean section or induction, if needed) 7. Identification and induction of mothers with 41 weeks of gestation or more 8. Skilled care at birth and immediate care for neonates 9. Basic emergency obstetric care 10. Comprehensive emergency obstetric care |  | *No* | *Yes* | *Antenatal Intrapartum* | *No* | *No* | *No* | *Yes* |
| **48** | 1. Antenatal Care 2. Pregnant women protected via intermittent preventive treatment of malaria (IPT) or sleeping under an insecticide-treated bed net (ITN) 3. SBA 4. Exclusive breastfeeding for the first six months of life 5. Vitamin A Supplementation 6. Insecticide treated bed nets or indoor residual spraying 7. Case management of diarrhoea (ORS) 8. Zinc for treatment of diarrhoea 9. Case management of pneumonia (oral antibiotics) 10. Case management of malaria |  | *No* | *Yes* | *Antenatal Intrapartum* | *Yes* | *No* | *Yes* | *No* |
| **49** | 1. Antenatal Care 2. Facility based delivery 3. Skilled birth attendance 4. Oral Rehydration Salt (ORS) 5. Case Management of Pneumonia 6. ITN 7. Artemisinin-based combination therapy |  | *No* | *Yes* | *Antenatal Intrapartum* | *Yes* | *Yes* | *Yes* | *No* |
| **50** | 1. Institutional delivery 2. Clean birth practices 3. Immediate assessment and stimulation 4. Labour and delivery management 5. Essential care for all women and immediate essential newborn care 6. Basic Emergency Obstetric Care 7. Comprehensive emergency obstetric care 8. Neonatal resuscitation 9. Antibiotics for preterm premature rupture of membranes 10. MgSO_4_ 11. Active management of the third stage of labour 12. Induction of labour for pregnancies lasting 41+ weeks 13. Preventive postnatal care 14. Kangaroo mother care 15. Case management of severe infection in neonates 16. PMTCT 17. Breastfeeding 18. Case management of pneumonia 19. Improved water source 20. Insecticide-treated materials or indoor residual spraying 21. Zinc for treatment 22. Oral rehydration solutions (ORS) 23. Antimalarials 24. Measles vaccine 25. Pneumococcal vaccine |  | *No* | *Yes* | *Antenatal Intrapartum Postpartum* | *Yes* | *No* | *Yes* | *No* |
| **51** | 1. Balanced energy supplementation 2. Diabetes case management 3. Hypertensive disorder case management 4. Intermittent preventive treatment of malaria during pregnancy 5. MgSO_4_ management of pre-eclampsia 6. Multiple micronutrient supplementation in pregnancy 7. Syphilis detection and treatment 8. Tetanus toxoid vaccination 9. Childbirth period Induction of labour for pregnancies lasting 41+weeks (tertiary care) 10. Antibiotics for pPROM 11. Active management of the third stage of labour 12. Clean birth practices 13. Labour and delivery management 14. Immediate assessment and stimulation of neonate 15. Neonatal resuscitation (facility-based) 16. Breastfeeding promotion 17. Measles vaccine 18. Meningococcal vaccine 19. Pentavalent vaccine 20. Pneumococcal vaccine 21. Rotavirus vaccine 22. Chlorhexidine 23. Clean postnatal practices 24. Hand washing with soap 25. ITN/IRS: households protected from malaria 26. education only about appropriate complementary feeding 27. Supplemental food and education for children ages 6–23 months from food insecure households 28. Vitamin A supplementation 29. Antibiotics for treatment of dysentery 30. Case management of neonatal prematurity including kangaroo mother care 31. Case management of neonatal sepsis/pneumonia with injectable antibiotics 32. Maternal sepsis case management 33. Oral antibiotics for pneumonia 34. Oral rehydration solution 35. Treatment for severe acute malnutrition with food supplementation 36. Treatment of malaria with artemisinin compounds 37. Vitamin A for treatment of measles 38. Zinc for treatment of diarrhoea 39. PMTCT 40. Cotrimoxazole 41. Antiretroviral therapy (ART) 42. Family planning |  | *No* | *Yes* | *Antenatal Intrapartum Postpartum* | *Yes* | *Yes* | *Yes* | *Yes* |
| **52** | 1. Antimalarial treatment 2. Skilled attendant at birth 3. Use of improved sanitation facilities 4. Use of improved drinking water sources 5. Institutional delivery 6. Care seeking for pneumonia 7. Hygienic disposal of children’s stools 8. Exclusive breastfeeding (1–5 months) 9. Oral rehydration solution 10. Antenatal care (at least four visits) 11. Exclusive breastfeeding (<6 months) 12. Neonatal tetanus protection 13. Exclusive breastfeeding (0–1 month) 14. Contraceptive prevalence 15. Early initiation of breastfeeding 16. Antenatal care (at least one visit) 17. Measles immunisation 18. Need for family planning satisfied 19. Three doses of combined diphtheria–pertussis–tetanus vaccine immunisation 20. Vitamin A supplementation 21. Use of water connection in the home 22. CS 23. Three doses of Haemophilus influenzae serotype b immunisation 24. Postnatal care for mothers 25. Artemisinin-combination treatment for malaria case management 26. Household ownership of insecticide-treated nets 27. Use of insecticide-treated nets by pregnant women 28. Use of insecticide-treated nets 29. Intermittent preventive treatment for malaria during pregnancy |  | *No* | *Yes* | *Antenatal Intrapartum Postpartum* | *Yes* | *Yes* | *Yes* | *Yes* |
| **53** | 1. Family planning demand satisfied 2. ANC (minimum one visit) 3. ANC (minimum four visits) 4. Tetanus (minimum doses) 5. Skilled delivery (skilled birth attendant) 6. Early initiation of breastfeeding 7. Exclusive breastfeeding 8. DPT3 vaccination 9. Measles vaccination 10. Care seeking for pneumonia 11. Diarrhoea treatment (oral rehydration solution) 12. Improved water sources 13. Improved sanitation 14. Hib vaccine 15. Reduction of stunting 16. Reduction of wasting 17. Oral antibiotics | *Authors report that “All available coverage indicators were recalculated using standard Countdown definitions or LiST definitions” however not all interventions are listed. Interventions explicitly within the manuscript have been extracted.* | *No* | *No* | *N/A* | *Yes* | *No* | *Yes* | *No* |
| **54** | 1. Antenatal care (1+) 2. Antenatal care (4+) 3. Any breastfeeding (12-23m infant age) 4. Any breastfeeding (6-11m infant age) 5. Care seeking for pneumonia 6. Child slept under an ITN 7. C-section 8. Demand for FP satisfied 9. DPT3 Penta3 immunization 10. Early initiation of breastfeeding 11. Exclusive breastfeeding (<1m infant age) 12. Exclusive breastfeeding (0-5m infant age) 13. Exclusive breastfeeding (1-5m infant age) 14. Facility delivery (clinic and hospital) 15. Hib immunization (3 doses) 16. Household ITN and insect repellent spray 17. Hygienic disposal of children's stools 18. Improved sanitation (can be shared) 19. Improved sanitation (cannot be shared) 20. Improved water source 21. Intermittent preventative treatment in pregnancy (for malaria) 22. Iron folate supplementation 23. Malaria treatment - Artemisinin 24. Malaria treatment (Antimalarials within 48 hours) 25. Malaria treatment (first line) 26. Measles immunization 27. Neonatal tetanus protection 28. ORS - oral rehydration solution 29. ORS + continued feeding 30. Partial breastfeeding (<1m infant age) 31. Partial breastfeeding (1-5m infant age) 32. Postnatal care for infant 33. Postnatal care for mothers 34. Predominant breastfeeding (<1m infant age) 35. Predominant breastfeeding (1-5m infant age) 36. Skilled birth attendance 37. Vitamin A supplementation 38. Water connection in the home | *.* | *No* | *Yes* | *Antenatal Intrapartum* | *Yes* | *No* | *Yes* | *No* |
| **55** | 1. Safe abortion services 2. Post abortion case management 3. Ectopic pregnancy case management 4. Folic acid supplementation or fortification 5. Syphilis detection and treatment 6. Tetanus toxoid 7. MgSO_4_ management of pre-eclampsia 8. Diabetes screening and management 9. Hypertensive disease case management 10. Malaria case management 11. Screening for foetal growth restriction and appropriate management 12. IPTp: pregnant women protected via intermittent preventive treatment of malaria during pregnancy or by sleeping under an ITN 13. Calcium supplementation 14. Protein energy supplementation 15. Multiple micronutrient supplementation 16. Antibiotics for PROM 17. Labour and delivery management 18. Clean birth practices at home 19. Antenatal corticosteroids for preterm labour 20. Immediate assessment and stimulation 21. Neonatal resuscitation 22. Active management of 3rd stage of labour 23. MgSO4 management of eclampsia 24. Induction of labour to prevent births at or beyond 41 completed weeks 25. Preventive postnatal care (healthy practices and illness detection) 26. Thermal care 27. Kangaroo mother care 28. Chlorhexidine 29. Case management of severe neonatal infection 30. Full supportive care for prematurity 31. Maternal Sepsis case management 32. ORS 33. Zinc for treatment of diarrhoea 34. Antibiotics for dysentery 35. Case management of pneumonia 36. Insecticide treated materials or indoor residual spraying 37. Therapeutic feeding for severe wasting 38. Treatment of moderate acute malnutrition 39. Exclusive breastfeeding till 6 months 40. Complementary feeding education and supplementation 41. Vitamin A supplementation 42. Zinc supplementation 43. Hib vaccine 44. Measles vaccine 45. DPT vaccination 46. Rotavirus vaccine 47. Pneumococcal vaccine 48. Use of improved water source within 30 minutes 49. Improved sanitation - utilization of latrines or toilets 50. Hand washing with soap 51. Hygienic disposal of children's stools |  | *No* | *Yes* | *Antenatal Intrapartum Postpartum* | *Yes* | *Yes* | *Yes* | *Yes* |
| **56** | 1. Contraceptive use 2. Folic acid supplementation/fortification 3. Safe abortion services 4. Post abortion case management 5. BEmOC facility 6. Ectopic pregnancy case management 7. Antenatal care - 4 or more ANC visits 8. TT - Tetanus toxoid vaccination 9. Intermittent preventative treatment in pregnancy for malaria 10. Syphilis detection and treatment 11. Calcium supplementation 12. Multiple micronutrients 13. Iron folate supplementation 14. Balanced energy supplementation 15. Hypertensive disease case management 16. Diabetes case management 17. Malaria case management 18. Magnesium sulphate Management of pre-eclampsia 19. FGR detection and management 20. PMTCT of HIV (including breastfeeding choices) 21. Facility delivery 22. Skilled birth attendance 23. Clean birth practices 24. Immediate assessment and stimulation 25. Labour and delivery management 26. Neonatal resuscitation 27. Antenatal corticosteroids for preterm labour 28. Antibiotics for preterm premature rupture of membranes 29. Magnesium sulphate management of eclampsia 30. AMTSL 31. Induction of labour for pregnancies 32. Thermal care 33. Clean postnatal practices 34. Chlorhexidine 35. Exclusive breastfeeding for first 6 months of life 36. Complementary feeding—education only 37. Complementary feeding--supplementation and education 38. Vitamin A supplementation 39. Zinc supplementation 40. Improved water source 41. Water connection in the home 42. Improved sanitation - Utilization of latrines or toilets 43. Hand washing with soap 44. Hygienic disposal of children's stools 45. Ownership of insecticide treated nets (ITN/LLIN) 46. BCG vaccine 47. Polio vaccine 48. DPT vaccine 49. Hib vaccine 50. Hepatitis B vaccine 51. Pneumococcal vaccine 52. Rotavirus vaccine 53. Measles vaccine 54. Maternal Sepsis case management 55. Kangaroo mother care 56. ORS 57. Case management of severe neonatal infection 58. Oral antibiotics 59. Injectable antibiotics 60. Full supportive care 61. Antibiotics - for treatment of dysentery 62. Zinc 63. Care-seeking for pneumonia 64. Vitamin A 65. Antimalarials – Artemisinin compounds for malaria 66. Therapeutic feeding - for severe wasting 67. Treatment for moderate acute malnutrition 68. Cotrimoxazole Coverage 69. ART Coverage |  | *No* | *Yes* | *Antenatal Intrapartum Postpartum* | *Yes* | *Yes* | *Yes* | *Yes* |
| **57** | 1. Change in wasting prevalence 2. ITN/Insect repelling spray 3. ACTs for treatment of malaria 4. Oral antibiotics for pneumonia 5. Changes in breastfeeding 6. Measles vaccine 7. Labour and delivery management 8. Change in stunting prevalence 9. PMTCT 10. Full supportive care, sepsis/pneumonia 11. Changes in breastfeeding 12. Full supportive care for neonatal sepsis/pneumonia 13. Tetanus toxoid vaccination 14. Neonatal resuscitation 15. Clean birth practices 16. Thermal care 17. Immediate assessment and stimulation 18. Antibiotics for pPROM 19. Oral antibiotics for neonatal sepsis/pneumonia 20. Active management of the third stage of labour 21. MgSO_4_ management of eclampsia 22. Contraceptive use 23. Hypertensive disorder case management 24. Malaria case management 25. IPTp 26. Syphilis detection and treatment 27. Micronutrient supplementation (iron and multiple micronutrients) 28. Maternal age and birth order 29. Birth intervals 30. Vitamin A supplementation 31. Improved water source 32. Water connection in the home 33. Improved sanitation - Utilization of latrines or toilets 34. Hygienic disposal of children's stools 35. DPT vaccine 36. H. influenzae b vaccine 37. Measles vaccine 38. Injectable antibiotics for neonatal sepsis/pneumonia 39. ORS 40. Cotrimoxazole | *Authors suggest 55 interventions were modelled but only 41 listed in the text and extracted here* | *No* | *Yes* | *Antenatal Intrapartum* | *Yes* | *Yes* | *Yes* | *No* |
| **58** | 1. ACT 2. IPTp 3. ITN 4. Stunting and wasting 5. Breast feeding 6. Vitamin A supplementation and measles treatment 7. Iron and multiple micronutrient 8. Hib vaccine 9. DPT vaccine 10. PCV vaccine 11. Rota vaccine 12. Measles vaccine 13. Hygienic disposal of stool 14. Improved sanitation 15. Labour and delivery management 16. Neonatal resuscitation 17. Clean post-natal 18. Clean birth 19. Immediate stimulation, 20. Antibiotic for PROM 21. PMTCT 22. ART 23. Cotrimoxazole 24. Maternal birth order and birth intervals 25. Case management of sepsis and pneumonia 26. Zinc for treatment of diarrhoea 27. Case management for premature babies | *Unclear if results reported for interventions reflect all modelled interventions (variation with the ‘indicator list’)* | *No* | *Yes* | *Antenatal Intrapartum Postpartum* | *Yes* | *No* | *Yes* | *No* |
| **59** | 1. Exclusive Breastfeeding for the first six months of life 2. Complementary feeding (6-9 months) 3. Vitamin A supplementation (one dose) 4. Measles immunization coverage 5. Diphtheria, pertussis, and tetanus immunization coverage (3 doses) 6. Oral rehydration salts 7. Household ownership of insecticide-treated nets 8. Care seeking for pneumonia 9. Care seeking for fever and cough illness 10. Early initiation of breastfeeding 11. Antenatal Care (at least one visit) 12. Antenatal Care (four or more visits) 13. Neonatal tetanus protection 14. Skilled attendant at birth 15. Reduction in stunting 16. Reduction in wasting | *Not clear which interventions were increased in LiST – extracted from supplementary material.* | *No* | *Yes* | *Antenatal Intrapartum* | *Yes* | *No* | *Yes* | *No* |
| **60** | 1. Contraception 2. Medical abortion 3. ANC 4. SBA for home births 5. Clean delivery 6. Hemorrhage management 7. Facility births 8. Non EmONC services 9. AMTSL 10. Partograph 11. Assisted delivery 12. Hypertension management 13. Sepsis management 14. Ectopic pregnancy management: 15. BEmONC services 16. CEmONC services 17. Quality of care 18. Referral 19. Transport 20. Targeted transfers | *N/A* | *No* | *Yes* | *Antenatal*  *Intrapartum*  *Postpartum* | *No* | *Yes* | *No* | *No* |

*Data extraction form part C – Model characteristics*

| **Study N^o^** | **Model name or description** | **Was the model developed for this study?** | **Is a model diagram included with study?** | **Is feedback in the model system linear or non-linear?** | **Is the model stochastic or deterministic?** | **The model is individual- or population-based?** | **Are changes modelled according to calendar time or age?** |
| --- | --- | --- | --- | --- | --- | --- | --- |
| **1** | *Individual-based dynamic microsimulation model* | *Yes* | *Yes* | *Linear* | *Stochastic* | *Individual* | *Calendar* |
| **2** | *The Lives Saved Tool (LiST)* | *No* | *No* | *Linear* | *Deterministic* | *Population* | *Calendar* |
| **3** | *Monte-Carlo simulation* using a decision tree structure | *Yes* | *No* | *Linear* | *Stochastic* | *Population* | *Calendar* |
| **4** | *LiST* | *No* | *No* | *Linear* | *Deterministic* | *Population* | *Calendar* |
| **5** | *Systems dynamics model/ Quantitative simulation model* | *Yes* | *Yes* | *Non-linear* | *Deterministic* | *Population* | *Calendar* |
| **6** | *Agent-based simulation model* | *Yes* | *No* | *Linear* | *Stochastic* | *Individual* | *Age* |
| **7** | *Mathematical model* | *Yes* | *Yes* | *Linear* | *Deterministic* | *Population* | *Age* |
| **8** | *Systems-levels and dynamic mathematical model* | *Yes* | *Yes* | *Non-linear* | *Stochastic* | *Individual* | *Calendar* |
| **9** | *LiST* | *No* | *No* | *Linear* | *Deterministic* | *Population* | *Calendar* |
| **10** | *LiST* | *No* | *No* | *Linear* | *Deterministic* | *Population* | *Calendar* |
| **11** | *LiST* | *No* | *No* | *Linear* | *Deterministic* | *Population* | *Calendar* |
| **12** | *Maternal and Neonatal Directed Assessment of Technology (MANDATE) model* | *No* | *Yes* | *Linear* | *Deterministic* | *Population* | *Age* |
| **13** | *LiST* | *No* | *No* | *Linear* | *Deterministic* | *Population* | *Calendar* |
| **14** | *LiST* | *No* | *No* | *Linear* | *Deterministic* | *Population* | *Calendar* |
| **15** | *LiST* | *Yes* | *Yes* | *Linear* | *Deterministic* | *Population* | *Age* |
| **16** | *LiST* | *No* | *No* | *Linear* | *Deterministic* | *Population* | *Calendar* |
| **17** | *MANDATE model* | *No* | *No* | *Linear* | *Deterministic* | *Population* | *Calendar* |
| **18** | *LiST* | *No* | *No* | *Linear* | *Deterministic* | *Population* | *Calendar* |
| **19** | *MANDATE model* | *No* | *No* | *Linear* | *Deterministic* | *Population* | *Calendar* |
| **20** | *MANDATE model* | *No* | *No* | *Linear* | *Deterministic* | *Population* | *Calendar* |
| **21** | *MANDATE model* | *No* | *No* | *Linear* | *Deterministic* | *Population* | *Calendar* |
| **22** | *MANDATE model* | *No* | *No* | *Linear* | *Deterministic* | *Population* | *Calendar* |
| **23** | *MANDATE model* | *No* | *No* | *Linear* | *Deterministic* | *Population* | *Calendar* |
| **24** | *LiST* | *No* | *No* | *Linear* | *Deterministic* | *Population* | *Calendar* |
| **25** | *Decision-analytic model* | *Yes* | *Yes* | *Linear* | *Deterministic* | *Population* | *Age* |
| **26** | *LiST* | *No* | *No* | *Linear* | *Deterministic* | *Population* | *Calendar* |
| **27** | *LiST* | *No* | *No* | *Linear* | *Deterministic* | *Population* | *Calendar* |
| **28** | *Decision tree model* | *Yes* | *Yes* | *Linear* | *Deterministic* | *Population* | *Calendar* |
| **29** | *Decision analytic model* | *Yes* | *Yes* | *Linear* | *Deterministic* | *Population* | *Calendar* |
| **30** | *LiST* | *No* | *No* | *Linear* | *Deterministic* | *Population* | *Calendar* |
| **31** | *LiST* | *No* | *No* | *Linear* | *Deterministic* | *Population* | *Calendar* |
| **32** | *Mixed micro-simulation markov model* | *Yes* | *Yes* | *Linear* | *Deterministic* | *Population* | *Age* |
| **33** | *Decision tree model* | *Yes* | *Yes* | *Linear* | *Deterministic* | *Population* | *Age* |
| **34** | *Multiplicative model* | *No* | *No* | *Linear* | *Deterministic* | *Population* | *Calendar* |
| **35** | *Cohort model* | *Yes* | *No* | *Linear* | *Deterministic* | *Population* | *Calendar* |
| **36** | *Decision tree model* | *Yes* | *Yes* | *Linear* | *Deterministic* | *Population* | *Calendar* |
| **37** | *LiST* | *No* | *Yes* | *Linear* | *Deterministic* | *Population* | *Calendar* |
| **38** | *LiST* | *No* | *No* | *Linear* | *Deterministic* | *Population* | *Calendar* |
| **39** | *MANDATE model* | *No* | *No* | *Linear* | *Deterministic* | *Population* | *Calendar* |
| **40** | *Monte-carlo simulation model* | *Yes* | *No* | *Linear* | *Deterministic* | *Population* | *Calendar* |
| **41** | *LiST* | *No* | *No* | *Linear* | *Deterministic* | *Population* | *Calendar* |
| **42** | *Global Maternal Health Policy Model* | *No* | *Yes* | *Linear* | *Deterministic* | *Individual* | *Calendar* |
| **43** | *LiST* | *No* | *No* | *Linear* | *Deterministic* | *Population* | *Calendar* |
| **44** | *LiST* | *No* | *No* | *Linear* | *Deterministic* | *Population* | *Calendar* |
| **45** | *LiST* | *No* | *No* | *Linear* | *Deterministic* | *Population* | *Calendar* |
| **46** | *LiST* | *No* | *No* | *Linear* | *Deterministic* | *Population* | *Calendar* |
| **47** | *LiST* | *No* | *No* | *Linear* | *Deterministic* | *Population* | *Calendar* |
| **48** | *LiST* | *No* | *No* | *Linear* | *Deterministic* | *Population* | *Calendar* |
| **49** | *LiST* | *No* | *No* | *Linear* | *Deterministic* | *Population* | *Calendar* |
| **50** | *LiST* | *No* | *No* | *Linear* | *Deterministic* | *Population* | *Calendar* |
| **51** | *LiST* | *No* | *No* | *Linear* | *Deterministic* | *Population* | *Calendar* |
| **52** | *LiST* | *No* | *No* | *Linear* | *Deterministic* | *Population* | *Calendar* |
| **53** | *LiST* | *No* | *No* | *Linear* | *Deterministic* | *Population* | *Calendar* |
| **54** | *LiST* | *No* | *No* | *Linear* | *Deterministic* | *Population* | *Calendar* |
| **55** | *LiST* | *No* | *No* | *Linear* | *Deterministic* | *Population* | *Calendar* |
| **56** | *LiST* | *No* | *No* | *Linear* | *Deterministic* | *Population* | *Calendar* |
| **57** | *LiST* | *No* | *No* | *Linear* | *Deterministic* | *Population* | *Calendar* |
| **58** | *LiST* | *No* | *No* | *Linear* | *Deterministic* | *Population* | *Calendar* |
| **59** | *LiST* | *No* | *No* | *Linear* | *Deterministic* | *Population* | *Calendar* |
| **60** | *Microsimulation model* | *Yes* | *No* | *Linear* | *Stochastic* | *Individual* | *Calendar* |

*Data extraction form part D – Analyses characteristics*

As with modelled interventions, study outcomes were copied verbatim from included studies. Reported in this table are study outcomes which can reasonably be defined as ‘health-related’. This includes estimates of cost-effectiveness which require evaluation of intervention impact on a health outcome.

| **Study**  **N^o^** | **Reported health-related study outcomes** | **Maternal mortality outcome?** | **Maternal Morbidity outcome?** | **Maternal DALYs outcome?** | **Neonatal mortality outcome?** | **Neonatal morbidity outcome?** | **Neonatal DALYs outcome?** | **Stillbirth outcome?** | **Outcomes reported by country?** | **Sensitivity analysis?** | **Analysis time horizon?** |
| --- | --- | --- | --- | --- | --- | --- | --- | --- | --- | --- | --- |
| **1** | 1. DALYS averted relative to baseline per 100,000 live births 2. Number treated per 100,000 live births 3. Total cost (2021 USD) per 100,000 live births 4. Incremental Cost Effectiveness Ratio (ICER) (2021 USD per DALY averted) relative to baseline | *No* | *No* | *No* | *No* | *No* | *Yes* | *No* | *Yes* | *Yes* | *2 years* |
| **2** | 1. Maternal deaths averted 2. Stillbirths averted 3. Child deaths averted 4. DALYs averted 5. Total cost per DALY averted 6. Total cost per death averted | *Yes* | *No* | *Yes* | *Yes* | *No* | *Yes* | *Yes* | *N/A* | *Yes* | *10 years* |
| **3** | 1. Total newborns treated over 10 years 2. Total number of newborns effectively treated over 10 years 3. Average cost per newborn effectively treated 4. Number of newborns effectively treated per 1,000,000 Kenyan shillings | *No* | *No* | *No* | *No* | *Yes* | *No* | *No* | *N/A* | *Yes* | *10 years* |
| **4** | 1. Average cost effectiveness ratio 2. Healthy Life Years per 1 mill population | *No* | *No* | *Yes* | *No* | *No* | *Yes* | *No* | *No* | *Yes* | *100 years* |
| **5** | 1. Maternal and neonatal healthcare awareness 2. Health delivery fraction 3. Neonatal dying rate 4. Fraction attending four antenatal care visits 5. Percentage decrease in neonatal dying rate (compared to base case) | *No* | *No* | *No* | *Yes* | *No* | *No* | *No* | *N/A* | *Yes* | *21 years* |
| **6** | 1. Neonatal mortality decreases per 1000 live birth 2. Women travelling further for care (%) 3. Mean increased travel distance, km 4. Utility decrement (km required to overcome) 5. Deliveries by CS, % | *No* | *No* | *No* | *Yes* | *No* | *No* | *No* | *N/A* | *No* | *1 delivery* |
| **7** | 1. Estimated reduction in deaths from postpartum haemorrhage and sepsis | *Yes* | *No* | *No* | *No* | *No* | *No* | *No* | *N/A* | *No* | *1 delivery* |
| **8** | 1. Maternal mortality | *Yes* | *No* | *No* | *No* | *No* | *No* | *No* | *N/A* | *No* | *3 months* |
| **9** | 1. Stillbirths averted 2. Maternal Lives Saved 3. Newborn lives saved 4. ICER | *Yes* | *No* | *No* | *Yes* | *No* | *No* | *Yes* | *N/A* | *No* | *15 years* |
| **10** | 1. DALYs averted (millions) 2. ICER (US$ per DALY averted) | *No* | *No* | *Yes* | *No* | *No* | *Yes* | *No* | *N/A* | *Yes* | *1 year* |
| **11** | 1. Stillbirths averted 2. Maternal Lives Saved 3. Newborn lives saved 4. Child lives saved 5. Life years gained 6. ICER | *Yes* | *No* | *No* | *Yes* | *No* | *No* | *Yes* | *Yes* | *Yes* | *1 year* |
| **12** | 1. Prevalence of PPH 2. PPH cases prevented by Tranexamic acid 3. Prevalence of severe PPH 4. Severe PPH cases prevented by Tranexamic acid 5. Deaths from PPH 6. PPH deaths prevented by Tranexamic acid vs Baseline | *Yes* | *Yes* | *No* | *No* | *No* | *No* | *No* | *No* | *No* | *1 delivery* |
| **13** | 1. Child lives saved 2. Maternal lives saved 3. Total child deaths | *Yes* | *No* | *No* | *Yes* | *No* | *No* | *No* | *N/A* | *No* | *15 years* |
| **14** | 1. Maternal lives saved 2. Incremental costs per DALY averted | *Yes* | *No* | *Yes* | *No* | *No* | *No* | *No* | *N/A* | *Yes* | *5 years* |
| **15** | 1. DALYs averted 2. Infant deaths averted 3. Maternal deaths averted 4. Skilled Births 5. Early ANC initiation 6. ICER | *Yes* | *No* | *Yes* | *Yes* | *No* | *Yes* | *Yes* | *N/A* | *Yes* | *1 pregnancy and delivery* |
| **16** | 1. Under 5 mortality rate (U5MR) 2. Infant mortality rate (IMR) 3. NMR 4. MMR | *Yes* | *No* | *No* | *Yes* | *No* | *No* | *No* | *N/A* | *No* | *15 years* |
| **17** | 1. Intrapartum related mortality 2. Lives saved 3. Cases of intrapartum related events 4. Cases of ongoing injury from intrapartum related events 5. Neonatal deaths from intrapartum related events 6. Neonatal deaths from ongoing intrapartum related injury 7. Total intrapartum related neonatal mortality 8. Intrapartum related mortality rate (per 1,000 live births) 9. Neonatal lives saved | *No* | *No* | *No* | *Yes* | *Yes* | *No* | *No* | *No* | *No* | *1 year* |
| **18** | 1. Neonatal deaths averted | *No* | *No* | *No* | *Yes* | *No* | *No* | *No* | *Yes* | *No* | *1 year* |
| **19** | 1. Maternal lives saved 2. Cases of severe haemorrhage 3. Number of surgeries | *Yes* | *Yes* | *No* | *No* | *No* | *No* | *No* | *No* | *No* | *1 year* |
| **20** | 1. Neonatal deaths 2. Neonatal deaths prevented from current level of care | *No* | *No* | *No* | *Yes* | *No* | *No* | *No* | *No* | *No* | *1 year* |
| **21** | 1. Neonatal deaths due to respiratory distress syndrome 2. Neonatal deaths due to intraventricular hemorrhage 3. Neonatal deaths due to necrotizing enterocolitis 4. Neonatal deaths due to preterm sepsis 5. Neonatal deaths due to preterm birth asphyxia 6. Neonatal deaths due to other prematurity 7. Preterm deaths prevented compared with current level of care | *No* | *No* | *No* | *Yes* | *No* | *No* | *No* | *No* | *No* | *1 year* |
| **22** | 1. Maternal deaths 2. Lives saved | *Yes* | *No* | *No* | *No* | *No* | *No* | *No* | *No* | *No* | *1 year* |
| **23** | 1. Stillbirths 2. Stillbirths prevented | *No* | *No* | *No* | *No* | *No* | *No* | *Yes* | *No* | *No* | *1 year* |
| **24** | 1. Maternal, newborn and child deaths averted | *Yes* | *No* | *No* | *Yes* | *No* | *No* | *No* | *No* | *No* | *15 years* |
| **25** | 1. Lifetime DALYs per patient 2. Cost per DALY Averted | *No* | *No* | *Yes* | *No* | *No* | *No* | *No* | *N/A* | *Yes* | *Lifetime horizon* |
| **26** | 1. Maternal lives saved 2. Child lives saved 3. Cost per life saved | *Yes* | *No* | *No* | *Yes* | *No* | *No* | *No* | *N/A* | *No* | *5 years* |
| **27** | 1. U5MR 2. NMR 3. MMR 4. Child lives saved 5. Newborn lives saved 6. Maternal lives saved 7. Incremental costs 8. Life years gained 9. Cost (US$)/LY gained | *Yes* | *No* | *No* | *Yes* | *No* | *No* | *No* | *N/A* | *No* | *5 years* |
| **28** | 1. ICER 2. DALYs averted 3. cost/DALY | *No* | *No* | *No* | *No* | *No* | *Yes* | *No* | *N/A* | *Yes* | *6 months* |
| **29** | 1. PPH cases averted 2. PPH related maternal deaths 3. ICER | *Yes* | *Yes* | *No* | *No* | *No* | *No* | *No* | *Yes* | *Yes* | *1 year* |
| **30** | 1. Under 5 child mortality 2. Diarrhoea specific mortality 3. Pneumonia specific mortality 4. Measles-specific mortality 5. Rate of breastfeeding 6. Rate of stunting 7. Rate of wasting 8. Cause specific child mortality 9. Maternal mortality | *Yes* | *No* | *No* | *Yes* | *Yes* | *No* | *No* | *No* | *No* | *1 year* |
| **31** | 1. Maternal deaths averted 2. Foetal deaths averted 3. Neonatal Deaths averted 4. Total deaths averted 5. Cost per death averted 6. Deaths averted with scale-up of midwifery | *Yes* | *No* | *No* | *Yes* | *No* | *No* | *Yes* | *No* | *No* | *3 years* |
| **32** | 1. Cost per DALY averted | *No* | *No* | *No* | *No* | *No* | *Yes* | *No* | *N/A* | *Yes* | *Lifetime horizon* |
| **33** | 1. Lives saved by exclusive breastfeeding 2. Lives saved by replacement feeding | *No* | *No* | *No* | *Yes* | *No* | *No* | *No* | *Yes* | *No* | *24 months of life* |
| **34** | 1. Deaths Averted | *No* | *No* | *No* | *Yes* | *No* | *No* | *No* | *No* | *Yes* | *1 year* |
| **35** | 1. Severe PPH cases 2. Severe PPH averted 3. Incremental cost-effectiveness ratio | *No* | *Yes* | *No* | *No* | *No* | *No* | *No* | *N/A* | *Yes* | *1 year* |
| **36** | 1. Maternal lives saved per year | *Yes* | *No* | *No* | *No* | *No* | *No* | *No* | *Yes* | *Yes* | *1 year* |
| **37** | 1. Maternal deaths 2. Stillbirth 3. Neonatal deaths | *Yes* | *No* | *No* | *Yes* | *No* | *No* | *Yes* | *No* | *Yes* | *4 years* |
| **38** | 1. Maternal deaths and maternal deaths averted 2. Neonatal deaths and neonatal deaths averted 3. Stillbirths and stillbirths averted | *Yes* | *No* | *No* | *Yes* | *No* | *No* | *Yes* | *No* | *No* | *15 years* |
| **39** | 1. % Reductions in (maternal) deaths with interventions from baseline of current care model | *Yes* | *No* | *No* | *No* | *No* | *No* | *No* | *No* | *No* | *1 year* |
| **40** | 1. Perinatal mortality, % change 2. Perinatal mortality, avoided deaths 3. Perinatal mortality, life years saved 4. Maternal mortality, % change 5. Maternal mortality, avoided deaths 6. Maternal mortality, life years saved | *Yes* | *No* | *No* | *Yes* | *No* | *No* | *Yes* | *N/A* | *No* | *1 year* |
| **41** | 1. Life years saved 2. Incremental life years saved 3. Incremental cost effectiveness ratio 4. ICER/Gross Domestic Product (GDP) per capita | *Yes* | *No* | *No* | *Yes* | *No* | *No* | *No* | *N/A* | *No* | *1 year* |
| **42** | 1. Reduction in maternal deaths (%) 2. Total fertility rates 3. Lifetime risk of maternal deaths 4. Proportionate mortality risk 5. Maternal deaths averted per 100,000 6. Cost effectiveness ratio (rounding) (US$ per years of life saved) | *Yes* | *No* | *No* | *No* | *No* | *No* | *No* | *N/A* | *Yes* | *12 years* |
| **43** | 1. MMR 2. Maternal lives saved 3. Child lives saved 4. Stillbirths prevented | *Yes* | *No* | *No* | *Yes* | *No* | *No* | *Yes* | *N/A* | *No* | *8 years* |
| **44** | 1. U5MR 2. NMR 3. Neonatal lives saved 4. Child lives saved 5. Maternal lives saved | *Yes* | *No* | *No* | *Yes* | *No* | *No* | *No* | *N/A* | *No* | *9 years* |
| **45** | 1. Maternal deaths averted 2. Neonatal deaths averted 3. Stillbirths averted 4. Child deaths averted | *Yes* | *No* | *No* | *Yes* | *No* | *No* | *Yes* | *Yes* | *Yes* | *4 years* |
| **46** | 1. Maternal deaths 2. Stillbirths 3. Neonatal deaths | *Yes* | *No* | *No* | *Yes* | *No* | *No* | *Yes* | *No* | *No* | *15 years* |
| **47** | 1. Stillbirths averted | *No* | *No* | *No* | *No* | *No* | *No* | *Yes* | *No* | *No* | *4 years* |
| **48** | 1. U5MR | *No* | *No* | *No* | *Yes* | *No* | *No* | *No* | *N/A* | *Yes* | *3 years* |
| **49** | 1. MMR 2. Maternal lives saved 3. U5MR 4. Under 5 lives saved | *Yes* | *No* | *No* | *Yes* | *No* | *No* | *No* | *N/A* | *No* | *4 years* |
| **50** | 1. U5MR 2. NMR 3. Deaths averted 4. Life expectancy at birth inequality in age at death | *No* | *No* | *No* | *Yes* | *No* | *No* | *No* | *N/A* | *No* | *4 years* |
| **51** | 1. U5MR 2. NMR 3. MMR 4. Stillbirth rate 5. Stunting prevalence 6. Intervention cost per death averted 7. Total cost per death averted | *Yes* | *No* | *No* | *Yes* | *Yes* | *No* | *Yes* | *No* | *No* | *13 years* |
| **52** | 1. U5MR 2. NMR | *No* | *No* | *No* | *Yes* | *No* | *No* | *No* | *Yes* | *No* | *25 years* |
| **53** | 1. Child deaths prevented 2. Neonatal deaths prevented | *No* | *No* | *No* | *Yes* | *Yes* | *No* | *No* | *N/A* | *No* | *11 years* |
| **54** | 1. Child lives saved by intervention per year | *No* | *No* | *No* | *Yes* | *Yes* | *No* | *No* | *N/A* | *No* | *13 years* |
| **55** | 1. Neonatal deaths averted 2. Under five deaths averted 3. Maternal deaths averted | *Yes* | *No* | *No* | *Yes* | *No* | *No* | *No* | *N/A* | *No* | *15 years* |
| **56** | 1. Under five deaths averted 2. U5MR | *No* | *No* | *No* | *Yes* | *No* | *No* | *No* | *N/A* | *No* | *12 years* |
| **57** | 1. U5MR 2. % reduction in under five mortality 3. Additional child lives saved 4. Neonatal mortality rate 5. % reduction in neonatal mortality 6. Additional neonatal lives saved 7. Proportion of total deaths by cause – child 8. Proportion of total deaths by cause – neonates 9. Proportion of total deaths by cause – mothers 10. MMR 11. % reduction in Maternal mortality 12. Additional Maternal lives saved | *Yes* | *No* | *No* | *Yes* | *No* | *No* | *No* | *N/A* | *No* | *18 years* |
| **58** | 1. Under five lives saved 2. Under five lives saved per year | *No* | *No* | *No* | *Yes* | *No* | *No* | *No* | *N/A* | *No* | *15 years* |
| **59** | 1. Child lives saved | *No* | *No* | *No* | *Yes* | *No* | *No* | *No* | *N/A* | *No* | *1 year* |
| **60** | 1. Total maternal deaths 2. MMR 3. Lifetime risk 4. Maternal deaths due to abortion 5. Maternal deaths due to hypertension 6. Maternal deaths due to haemorrhage 7. Maternal deaths due to sepsis 8. Maternal deaths due to obstructed labour 9. Maternal deaths due to other direct causes 10. Late maternal deaths 11. Indirect maternal deaths | *Yes* | *No* | *No* | *No* | *No* | *No* | *No* | *Yes* | *No* | *8 years* |

1. Interventions have been copied verbatim from papers in the first instance. Where appropriate clarity has been improved by adding additional details. [↑](#footnote-ref-1)
