# Supplementary material for: Mathematical modelling to estimate the impact of maternal and perinatal healthcare services and interventions on health in sub-Saharan Africa: A scoping review
Source: PLoS One. 2024 Dec 2;19(12):e0296540. doi: 10.1371/journal.pone.0296540 (PMC11611201; doi:10.1371/journal.pone.0296540)
Supplement: Attachment — Submitted filename: Response to reviewers comments.docx [file pone.0296540.s003.docx]

**Response to reviewers’ comments**

*Reviewer #1:*

*Thank for inviting me to the interesting scoping review by Joesph and colleagues on the mathematical models uses to estimate the impact of maternal and perinatal health intervention in Sub-Saharan Africa". I read and reviewed the scoping review with interest as it describes the different modeling exercises done in the last 50 years to describe the impact of scaling up the intervention on perinatal mortality outcomes. This is indeed an interesting paper, which provides what modeling were being done and which interventions were focused on. Most of the modelling that were done were based on the different clinical or population-based studies done to assess the effectiveness of the intervention. What this paper has shown it that lot of modelling had been published between 2015-2019, indicating the work in the end of the MDG period and start of the SDG period. Most of the modeling were done for interventions before birth (antenatal period) and intrapartum period (skilled birth attendance, neonatal resuscitation, magnesium sulfate). While the postnatal intervention modelling has not been done such as small and sick newborn care.*

*So, reading this scoping review, I have some major comments on the paper and some minor comments to consider.*

We would like to thank the reviewer for their review of our manuscript and for recognising the impact of this research and the key findings. Please find responses to the reviewers’ major and minor comments below.

Major comments:

**1. Since, this was a PhD project of the first author, was there any set protocol in place and registration of the scoping review done. There are several systematic review registration sites, such as Joanna Briggs Institute, Campbell collaboration, Prospero, research registry. Prospective scoping review protocol will always help reviewers to check any deviation in the review from that of the original intent. There can be deviation, for which amendment of the registration can be done. If this was not done, the transparency of the review, a time comes into scrutiny. The process needs to be provided in the method section.**

We thank the reviewer for this comment. For this review there is not a prospectively registered protocol that is publicly available. Whilst we recognise the importance of the prospective registration of systematic review protocols, within this study we have followed the methodological approach to scoping review conduct detailed by Levac et al. (2010). As highlighted in the introduction, this type of review is distinct from a traditional systematic review. Levac et al. (2010), whose framework for scoping review conduct builds on the seminal work of Arksey and O’Malley, describe the study selection phase of a scoping review as being highly iterative with reviewers returning to the search strategy, and amending the strategy if needed, to ensure the primary research question can be answered. This is methodologically very different from a systematic review and therefore does not lend itself to registration of a protocol (given that a changing search strategy is part of the review process). For example, the Prospero website does not accept scoping review protocols for preregistration (<https://www.crd.york.ac.uk/prospero/>). Further details on the differences between Systematic and Scoping Review approaches can been found in the paper by Munn (2018) cited below.

Importantly, we have sought to be transparent within the manuscript relating to how the search strategy was reviewed and changed during study conduct (i.e. the inclusion of the term ‘Lives Saved Tool’ as discussed below). Additionally, given that this review was conducted as part of the PhD, the initial search and study protocol was reviewed at multiple times during the length of the first authors PhD including MPhil to PhD upgrade, and by the first authors supervisors. Additionally this PhD has also been examined by external examiners.

Munn, Z., Peters, M.D.J., Stern, C. et al. Systematic review or scoping review? Guidance for authors when choosing between a systematic or scoping review approach. BMC Med Res Methodol 18, 143 (2018). https://doi.org/10.1186/s12874-018-0611-x

**2. The researcher in the objective set out to find "how have mathematical modelling been used to evaluate the delivery of maternal and perinatal health intervention" and in your third specific objective the research explain it "to critically discuss the use of the common modelling approach". The paper discusses on "what modelling exercise, where, and why". I do not understand the scoping review entailing the use of the modelling in policy practice. I might have misunderstood, but please clarify that this undertaking was what component of different mathematical modelling.**

We thank the reviewer for pointing this out and hope to clarify the study aim and objectives here. Through this review we did not seek to explore how models have practically been applied within the context of policy making within sub-Saharan Africa. Our review is focused on, as you highlight, the modelling methodologies which have been used within the academic literature and the interventions these methods have been used to evaluate. We sought to then, in the discussion, broadly discuss the strengths and limitations of these modelling methodologies critically. We agree that our aim should therefore be clarified and have now amended the studies primary aim to read “*What are the characteristics of mathematical models which have been applied within the academic literature to estimate the impact of the delivery of maternal and/or perinatal healthcare interventions or services on health-related outcomes in sub-Saharan Africa?”.* This has now been updated in the abstract and main body of the text and in the abstract.

**3. In the search strategy in page 7, lines 156 to 159, you mentioned, that you originally limited your search for Lives Saving Tools, was that your original idea of the scoping review of focusing on the LiST intervention only. I think your scoping review encompasses all mathematically modelling or did you start with List and expanded your mathematically model to other. This is the reason; I am requesting for a prospective registration of the study.**

Apologies for the confusion on this point. Please see our discussion on the iterative nature of the scoping review search methodology defined by Levac et al. in response to major comment 1 above. We conducted the initial search with the search terms defined in Table 1 as described in the manuscript. When reviewing the titles returned from this search, we identified there were many studies which used the Lives Saved Tool as a modelling methodology. Because the term “Lives Saved Tool” was not in the original search terms (and importantly does not include the word ‘model’) we amended our initial search strategy, by conducting an additional search, to ensure a comprehensive scoping of the literature had been conducted to answer the studies research question and that these highly relevant papers were included. As discussed, we believe this aligns closely with the iterative methodology of a scoping review defined by Levac et al.

**4. In the result section, using table 3. a time series presentation on the different modelling being done over the years using different color codes would be very interesting. You present in the table 2, the years of publication, an elaboration will be interesting. Moreover, the year of publication to the different modeling done for timing of intervention and type of intervention will be of value for two important reasons. Global perinatal health expanded in the MDG era with the focus first on the essential care for mothers and newborns and then focusing on high-risk care. The different years when different modeling by interventions were done will be of value to the reader. Your supplementary table 2 has all the ingredients to it.**

We believe that from the data presented in Table 3 (and Supplementary Table 2) it is apparent that most of the included papers modelled multiple interventions for women and neonates, and across the antenatal, intrapartum and postpartum period. We feel this suggest there is not a clear evolving picture of change over time. As such we will allow readers to review the relevant tables provided with the manuscript.

**5. The main component of your work is the analysis of the different mathematical modeling i.e table 4, the explanation of the different model structure especially the linear and non-linear feedback, the deterministic vs stochastic model needs explanation, as the model output varies especially due to these two factors. I think a good table where different models used linear vs non-linear, deterministic vs stochastic is highly recommended.**

Thank you for your comment. Within the text at lines 325-328 of the manuscript we define the difference between a linear and a non-linear model and similarly at lines 338-342 we define the difference between stochastic and deterministic models. We have defined these structural approaches at this point in the manuscript to provide the reader with more context relating to model structure. Additionally, at line 322 we reference the paper by Garnett et al. which defines these structural model components in detail. To improve clarity we have added Figure 1 which briefly summarises the model structural features we have included in the review.

**Minor comments:**

**Table 5 can be merged with table 2 or 3 or having a graphical presentation.**

Thank you. To reduce the number of tables in the manuscript we have now combined Tables 4 and 5. Table 4 now contains all information about the models and the model analyses conducted.

**Some minor comments, please do not provide or explain the details of different intervention such a between lines 268 to 279.**

Given the limited information in this comment we are unclear of the reviewer’s rationale for asking for this information to be removed from the manuscript. We feel that such information is contextually important and has been left in the text.

**Title is slight confusing, can you have " The mathematical models been used to evaluate the delivery of maternal and/or perinatal health interventions and their impact on health-related outcomes in sub96 Saharan Africa?”.**

We thank the reviewer for this suggestion which we have considered but we prefer to keep the existing title.

*Reviewer #2:*

*This is an interesting manuscript by Joseph and colleagues and can provide new literature on the different mathematical models used to project coverage and mortality.*

We would like to thank the reviewer for their review of our manuscript and for their comments which have responded to below.

*I have a few comments in place*
**- In the abstract, can you make it sub-titles with introduction, method, result and discussion. The term such as "Models were most applied to estimate impact" needs to be quantified in the result section. It would be good to provide which software's were used in different models, if possible, in the abstract.**

We thank the reviewer for their comment and have now added these subtitles to the abstract. Additionally, as suggested, we have now added additional quantifications to the results section of the abstract. Finally, we did not extract the specific software (e.g. R, STATA, Microsoft Excel) used for each of the included models and therefore cannot include this explicitly in the abstract or manuscript.

**-Can you provide the list of different mathematical models in the introduction section of main text**

We have now added a sentence in the introduction (line 64-68) which highlights some of the commonly applied models within health.

**-Can authors provide the policy and programmatic implication of this in the introduction section**

Within the introduction we highlight that mathematical modeling has a growing place in the development of health policy due to the number of complex policy questions which cannot be answered with experimentation (due to costs, ethics etc.) We go on to note that modelling, as a tool, therefore has the potential to inform policy changes that provide substantial health gains within maternal and newborn health in SSA due to persistently high mortality and address a wide variety of complex service-related policy questions. We summarise that despite evidence that models are being applied more widely in public health, there has been no formal evaluation of modelling within this field and region to understand the potential methodological gaps.

**-Why was this scoping review limited to Africa and not to Asia?**

This review is focused on sub-Saharan Africa as the PhD project to which this review contributed was focused on the development of a novel mathematical model for application in Malawi. We were therefore interested in exploring models within this field in the geographical context of the African region to contextualise this modelling work.

**- Was English language literature only searched or in other languages such as Western African countries use other European language.**

In lines 156-159we highlight that only English language papers were included. This is due to the first author only speaking the English language and because this review was conducted in the context of a PhD. We have now added a line to the limitations section of the paper to highlight this.

**- I think it would be good to see the comparison of the estimates provided by different mathematical model, as it provides the variance.**

Due to the heterogeneity of the models applied, the interventions evaluated, and the analyses conducted within the studies included in this review we do not see the merit in presenting the results of the individual studies included. Practically, this would be very difficult to achieve in a succinct and meaningful way and goes beyond the scope of this review. As outlined in the study aims, and in-keeping with the methodological approach of a scoping review described by Levac et al. and PRISMA-SCR, we sought to map the literature relating to the application of mathematical modelling approaches and characterise the models and their applications.
